# Characterization of two novel lytic bacteriophages having lysis potential against MDR avian pathogenic *Escherichia coli* strains of zoonotic potential

**DOI:** 10.1038/s41598-023-37176-z

**Published:** 2023-06-20

**Authors:** Sadia Sattar, Marc Bailie, Akasha Yaqoob, Sofia Khanum, Kaniz Fatima, Anees Ur Rehman Bin Altaf, Ibrar Ahmed, Syed Tahir Abbas Shah, Javeria Munawar, Quaratul Ain Zehra, Sajeela Daud, Ayesha Arshad, Kaleem Imdad, Sundus Javed, Amira Tariq, Nazish Bostan, Eric Altermann

**Affiliations:** 1 Molecular Virology Labs, Department of Biosciences, Comsats University Islamabad, Islamabad, 45550 Pakistan; 2 AgResearch, Palmerston North, 4410 New Zealand; 3 Alpha Genomics Private Limited, Islamabad, 45710 Pakistan; 4 Functional Genomics Lab, Department of Biosciences, Comsats University Islamabad, Islamabad, 45550 Pakistan; 5 Microbiology and Immunology Lab, Department of Biosciences, Comsats University Islamabad, Islamabad, 45550 Pakistan; 6 School of Veterinary Science Massey University Centre for Bioparticle Applications, Massey University, Palmerston North, 4472 New Zealand

**Keywords:** Microbiology, Bacteriophages

## Abstract

Avian pathogenic *E. coli* (APEC) is associated with local and systemic infections in poultry, ducks, turkeys, and many other avian species, leading to heavy economical losses. These APEC strains are presumed to possess zoonotic potential due to common virulence markers that can cause urinary tract infections in humans. The prophylactic use of antibiotics in the poultry sector has led to the rapid emergence of Multiple Drug Resistant (MDR) APEC strains that act as reservoirs and put human populations at risk. This calls for consideration of alternative strategies to decrease the bacterial load. Here, we report isolation, preliminary characterization, and genome analysis of two novel lytic phage species (*Escherichia* phage SKA49 and *Escherichia* phage SKA64) against MDR strain of APEC, QZJM25. Both phages were able to keep QZJM25 growth significantly less than the untreated bacterial control for approximately 18 h. The host range was tested against *Escherichia coli* strains of poultry and human UTI infections. SKA49 had a broader host range in contrast to SKA64. Both phages were stable at 37 °C only. Their genome analysis indicated their safety as no recombination, integration and host virulence genes were identified. Both these phages can be good candidates for control of APEC strains based on their lysis potential.

## Introduction

*Escherichia* coli can be broadly categorized into commensal and pathogenic *E. coli* based on its virulence. Pathogenic *E. coli* has two broad divisions Intestinal Pathogenic *Escherichia coli* (IPEC) and Extra-intestinal Pathogenic *Escherichia coli* (ExPEC)^1^. There are seven major phylogroups of *E. coli* isolates as illustrated by Clermont in 2012^2,3^. These groups include A, B1, B2, C, D, E and F. Major virulent Extra-intestinal pathogenic *E. coli* (ExPEC) strains belong to phylogroup B2 and D whereas most commensal strains are associated with group A or group B1^4,5^. Avian Pathogenic *E. coli* (APEC) a sub-division of ExP*EC*, is responsible for local and systemic infections in poultry such as colibacillosis . It is characterized by coligranuloma^6^, pericarditis, egg peritonitis, Airsacculitis, arthritis, life-threatening septicemia, salpingitis and perihepatitis^7^. APEC also affects ducks, turkeys, and other bird species. In turkeys, osteomyelitis complex^8^ and in chickens swollen head syndrome^9^ results from APEC infection . APEC infections outcome together with its treatment expenditures cost millions of dollars worldwide. According to an estimate, APEC affects nearly 30% of commercial flocks. Chicken of all ages e.g., young birds of 1–5 days as well as egg laying age are vulnerable to APEC infections. This case is especially true for chickens between 4 to 6 weeks old^1^. Such APEC strains survive in food chain and presence of common virulence markers with UPEC strains is indicative of their zoonotic potential^10^.

In developing countries like Pakistan, population density has placed a great pressure on animal rearing practices. To have maximum production in small places with poor housing, antibiotics are frequently prescribed, often in sub therapeutic doses to avoid infection and as growth promoters^11^. That results in the origin of multiple drug resistance (MDR) bacterial strains in animal sector. Presence of such MDR strains in food chain that possess common virulence markers as human Uro-pathogenic *E. coli* strains (UPEC) can lead to greater exposure of human population to these MDR strains with probable zoonotic potential and pose threat to human health resulting in regular outbreaks leaving little choice for physicians to treat them^12^ To control such MDR variants to non-infectious levels phage therapy has been a subject of interest from a long time^13,14^. Bacteriophages are natural killers of bacteria and are host specific unlike broad spectrum antibiotics that affect commensal bacterial populations along with pathogenic ones. They are abundant in almost every habitat^15^. In last two decades the use of lytic bacteriophages to control infections in burn sepsis patients and wound infections have given a new hope to control MDR bacteria^16–18^. Several studies have reported isolation and characterization of phages against APEC isolates as well^19,20^ A recent study has also reported potential of phages to reduce APEC load in vivo^21^ However, all this success majorly depends on discovery and detailed characterization of new phage species that can specifically target pathogenic bacteria. Phage must possess few basic characteristics for use as biocontrol or therapeutic agent such as being exclusively lytic (carrying no genes for lysogeny and recombination), possess good efficacy and safety, and should have pH and temperature stability for normal storage. Preferably they should be completely sequenced, and their genomes should be evaluated for presence of toxins and resistance markers. In addition, phages must have a good virulence potential against target bacteria. Testing a complete list of desired phage properties can be limiting due to financial and time constraints however, certain basic set of evaluations must be performed before using any phage^22–24^. In this context we designed this study to isolate, characterize and evaluate virulence potential and check stability of two novel lytic bacteriophages against MDR strain of *E. coli* of poultry origin. We also characterized phage genomes for presence of any toxins or virulence genes.

## Results

### Phylogenetic groups, virulence genes (VGs) and resistance markers in Escherichia coli strains

Samples were collected from chicken, and human ICU patients showing signs and symptoms of *E. coli* infection (See materials and methods). Out of 90 samples tested *E. coli* was detected in 46% (41/90) of isolates through a combination of biochemical and molecular identification protocols. Out of total, 43% (n = 26) isolates of poultry origin (*E. coli*p) while 50% (n = 15) of human urine samples tested positive for *E. coli* (*E. coli*H). These *E. coli* isolates were distributed to various phylogroups using the quadruplex PCR method developed by Clermont^2,3^. Phylogroup A (40%) was predominant in *E. coli*H, followed by phylogroups B2 (33.3%) and D (26.6%). In *E. coli*p isolates phylogroup D (34.6%) was most prevalent followed by B1 (23.1%), A (19.2%), Clade I (15.4%), F and B2 (3.8%). Phylogroups D and A were common to both *E. coli*p and *E. coli*H isolates. *fim*H and *ecp*A were the most common (100%) Virulence genes (VGs) in *E. coli*H isolates followed by *tra*T (80%), *pap*C (67%), *vat* (40%) and *omp*T (13%) whereas only one isolate was positive for *Stx1* and *Aggr* (6.7%). *E. coli*p isolates were all positive for *fim*H and *tra*T, followed by *ecp*A (96%), *pap*C (77%), *Aggr* (50%), *omp*T (39%) and *vat* (12%) gene. *vat*, *omp*T, *ecp*A, *papC*, *fim*H, and *tra*T were common to both poultry and human *E. coli* isolates (supplementary Table S1). These isolates were also resistant to at least one antibiotic from four classes of antibiotics tested and therefore classified as MDR with MAR indices ranging from 0.36 to 1 (supplementary Table S1). *E coli*p isolates depicted 100% resistance against *erythromycin*, *tetracycline*, and *ampicillin* followed by *streptomycin* and *sulphamethoxazole* (93%, 85%), *trimethoprim* (89%), *chloramphenicol* and *neomycin* (81%), *kanamycin* (70%) and least resistant against *cephalosporin*; *cefotaxime* (15%); Whereas *E coli*H isolates were 100% resistant to both *ampicillin* and *cefotaxime* followed by *erythromycin* (94%), *trimethoprim* and *neomycin* (82%), *nalidixic acid* and *tetracycline* (71%), *sulphamethoxazole* (65%), *streptomycin* (53%), *kanamycin* (41%) and *chloramphenicol* (35%) (supplementary Table S2). QZJM25 strain was further tested against 16 antibiotics including extended spectrum beta lactams and was resistant to 20 out of 27 antibiotics in total. This strain was resistant to 8 out of 10 beta lactam antibiotics tested, for the other two it had intermediate sensitivity. In addition, QZJM25 was resistant to at least one antibiotic from aminoglycosides, tetracyclines, quinolones, glycopeptides, macrolides, and sulphonamides therefore it was classified as muti-drug resistant (MDR). This strain was further confirmed by 16 s rRNA analysis (Macrogen). Due to its high resistance profile and presence of common virulence genes with human isolates this strain was selected for isolation and characterization of bacteriophages. Supplementary Table S3 gives the resistance profile of the host strain QZJM25. All primers used in this study for *E. coli* typing, pathotyping, and phylotyping are listed in supplementary Table S4.

### One step growth curve and stability of phages

**Figure 1:**
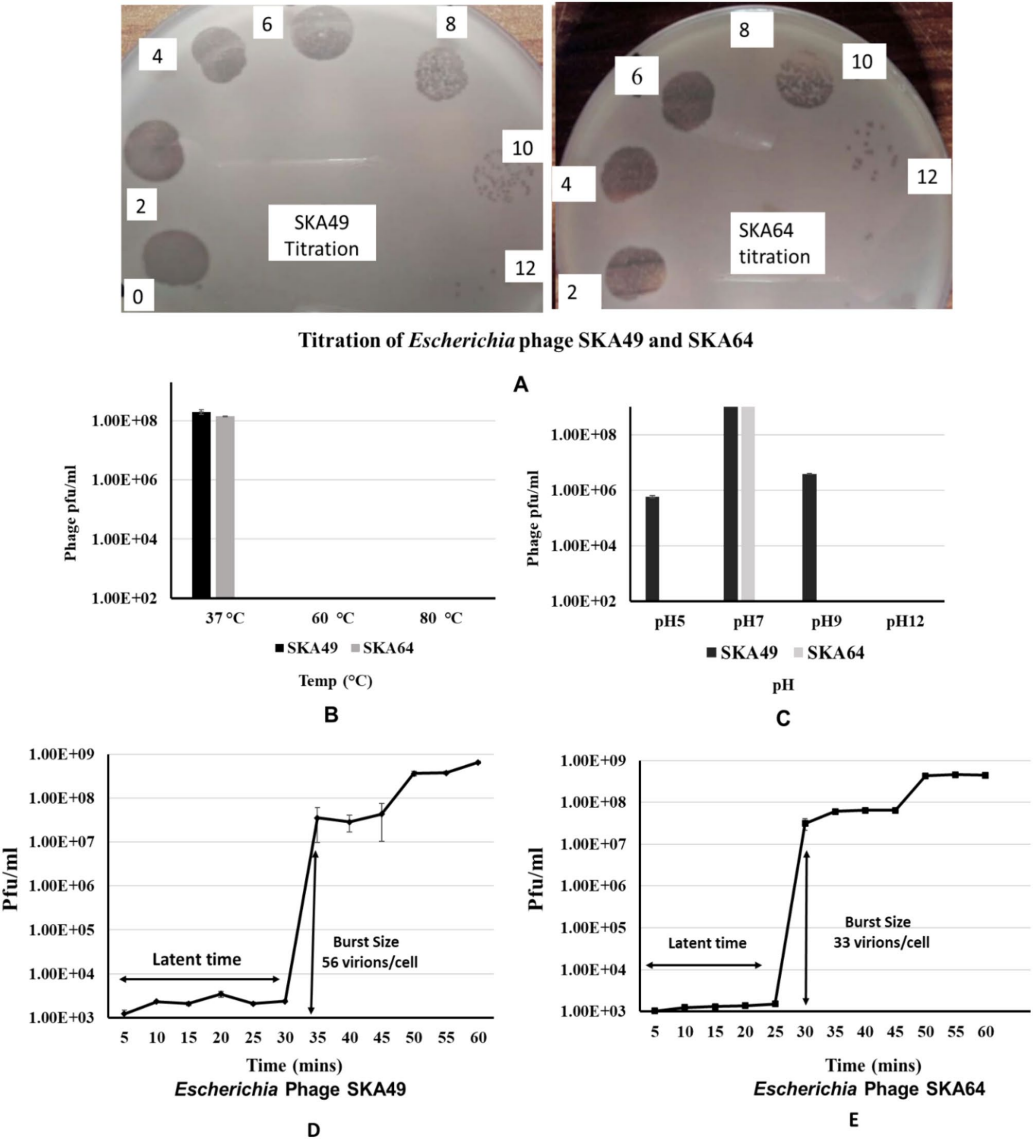
Characterization of *Escherichia* phage SKA49 and SKA64; (**A**) Titration and plaque type, (**B**) Temperature tolerance, (**C**) pH tolerance, and (**D**) SKA49 One step growth curve, (**E**) SKA64 One step growth curve.

*Escherichia* phage SKA49 and SKA64 were isolated from poultry tissue samples (broiler chicken) previously tested positive for presence of *Escherichia* Spp., using *Escherichia coli* strain QZJM25 (Gene Bank Acc. No OK086691) as host. Phages produced clear zones of lysis in titration indicating their lytic nature (Fig. 1A). Both SKA49 and SKA64 grew best at 37 °C giving highest titer of 1.8 × 10^9^ pfu / ml and 1.42 × 10^8^ respectively, however they were not infective at higher temperatures as no plaques were observed after incubation for 1 h at 60 °C, and 80 °C (Fig. 1B). SKA49 had better pH tolerance than SKA64, as it survived well at pH5 (5.83 × 10^5^ pfu/ml) and pH9 (3.93 × 10^6^ pfu/ml) in addition to pH7 (1.79 × 10^9^) albeit with significant (*p* = 0.0001) drop in titer. SKA64 on other hand was infective only at pH7 (2.8 × 10^9^) and no plaques were observed after incubation at pH5 and pH9. Neither phage survived at pH 12 (Fig. 1C). The burst size and latent period of both phages was calculated using a one-step growth curve as illustrated in materials and methods. The latent time for SKA49 was 35 min whereas it produced 56 virions /cell (± 7) (Fig. 1D). Whereas SKA64 has a 30 min latent period and burst size of 33 virions/cell (± 5) (Fig. 1E) (supplementary Table S5).

### QZJM25 growth reduction by SKA49 and SKA64

The ability of SKA49 and SKA64 phages to restrict growth of MDR APEC strain QZJM25 was determined at MOI1 and MOI100. Both MOIs worked well, and phages were able to restrict the growth of QZJM25 at background levels for 12 h. For SKA49 when MOI1 and MOI100 were compared the difference was statistically non-significant till 12 h indicating that increasing number of phages did not increase the bacterial lysis. From 14 to 18 h MOI100 was significantly more efficient in controlling growth than MOI1. Both MOI1 and 100 significantly controlled QZJM25 growth (0.0001) until 12 h after which the titer of QZJM25 increased but the difference was statistically significant till 18 h post inoculation (*p* = 0.001).

**Figure 2:**
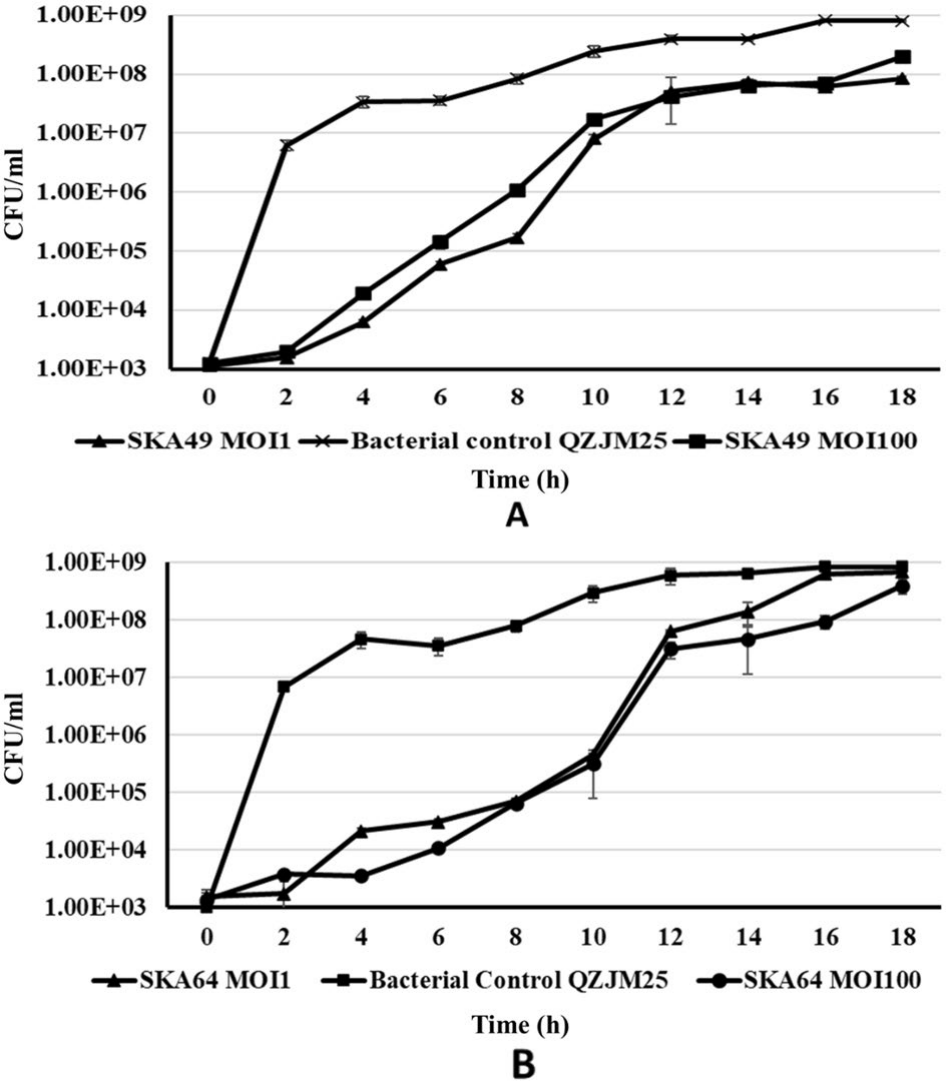
Bacterial Growth Reduction Assay in liquid cultures (**A**) *Escherichia* phage SKA49 and (**B**) *Escherichia* phage SKA64 at **MOI1** and **100**. *Escherichia coli* strain QZJM25 used as bacterial control without phages.

Likewise, SKA64 significantly restricted growth of QZJM25 at both MOI1 and 100 till 14h post infection keeping bacterial count less than QZJM25 control group (*p* = 0.008). From 16 to 18 h MOI100 was significantly more efficient in controlling QZJM25 growth than MOI1 (*p* = 0.02). (Fig. 2A,B). Time zero in Fig. 2A and, B represents time after adsorption of phages to host strain.

### Host range

The host range of both phages was tested on 36 isolates of *Escherichia* coli, isolated and characterized during this study in MVL using biochemical and molecular profiling. In total 21 isolates of poultry origin (*E. coli*p series), and 15 isolates of human origin (*E. coli*H series) were used to test SKA49 host range. In total 39% isolates (14 out of 36; 9 of poultry and 5 of human origin) were sensitive to SKA49 and produced clear zones of lysis, whereas 27% isolates allowed infection but not growth of phage as indicated by turbid zones. The remaining 35% isolates were completely resistant as no clear zones of lysis were produced. SKA64 host range was narrow as it infected only two poultry isolates out of 21 tested and produced turbid plaques on 3 *E. coli*H isolates with no titration results indicating it only infected and did not grow in these isolates (Table 1). However, both phages produced clear zones of lysis on QZJM25 and replicated with good titer.

**Table 1:** SKA49 and SKA64 host range as tested on field isolates of *Escherichia* coli.

Serial No	APEC *Escherichia coli* strains (isolated from poultry)	*Escherichia* phage SKA49	*Escherichia* phage SKA64
1	*E. coli* p 1	+	+
2	*E. coli* p 2	+ + +	–
3	*E. coli* p 6	–	–
4	*E. coli* p 7	+ + + +	–
5	*E. coli* p 19	–	–
6	*E. coli p 25 (QZJM25)	+ + + +	+ + + +
7	*E. coli* p 27	+ + + +	+ + + +
8	*E. coli* p 28	–	–
9	*E. coli* p 33	–	–
10	*E. coli* p 34	–	–
11	*E. coli* p 36	+ + +	–
12	*E. coli* p 39	+ + +	–
13	*E. coli* p 45	–	+ + +
14	*E. coli* p 46	+ +	–
15	*E. coli* p 47	+ + +	–
16	*E. coli* p 48	+ +	–
17	*E. coli* p 49	+	–
18	*E. coli* p 51	+	+
19	*E. coli* p 66	+ + +	+
20	*E. coli* p 68	+	+
21	*E. coli* p 69	+	–

Serial No	UPEC *Escherichia* coli (Isolated from human urine samples)	SKA49	SKA64
1	*E. coli* H1	–	+
2	*E. coli* H 2	–	–
3	*E. coli* H 3	+ + + +	–
4	*E. coli* H 5	+	+
5	*E. coli* H 6	–	–
6	*E. coli* H19	–	–
7	*E. coli* H 8	+ + +	–
8	*E. coli* H 9	+ + +	–
9	*E. coli* H 11	+ + +	–
10	*E. coli* H 12	–	–
11	*E. coli* H 13	+	+
12	*E. coli* H 27	+ + +	–
13	*E. coli* H 15	–	–
14	*E. coli* H16	+	–
15	*E. coli* H22	+	–

*E. coli* p (isolates of poultry origin), *E. coli* H (isolates of human origin), *Host strain for SKA49 and SKA64 isolation, (+ +  + +) clear lysis, (+ + +) Too numerous to count plaques, TNTC, (+ +) countable plaques ( +) light zone, (–) no lysis.

### Genome analysis of *Escherichia* phage SKA49

**Figure 3:**
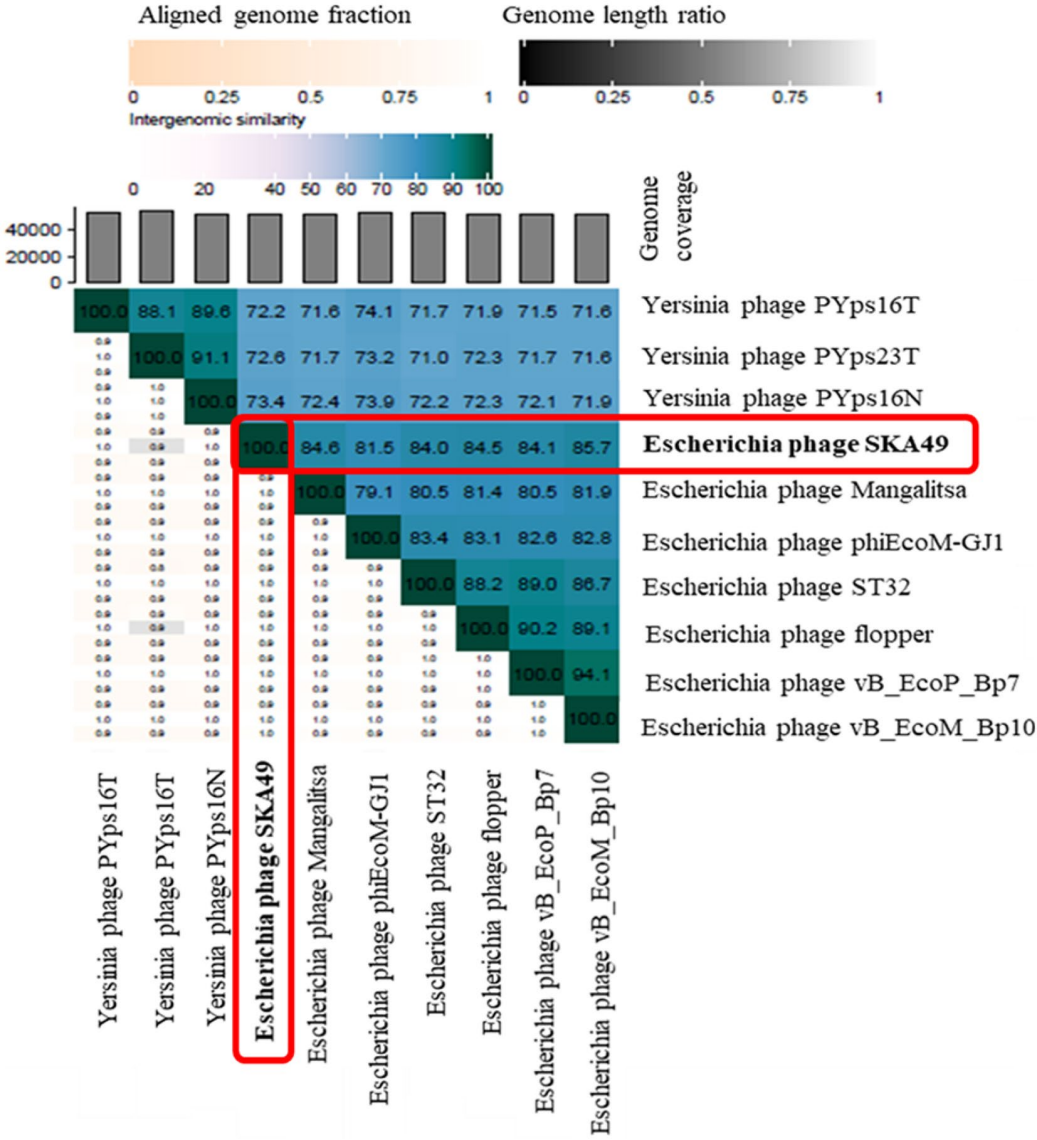
Comparative genome analysis of *Escherichia* phage SKA49 using VIRIDIC software (http://rhea.icbm.uni-oldenburg.de/VIRIDIC/) as per ICTV recommendations. The heatmap generated indicates the relationship of SKA49 with its close homologs in BLASTn and gives an accurate estimation of genomic similarity between phages.

SKA49 possesses a double stranded DNA genome of 51.548 Kilo base pairs with a GC content of 44%. When compared in nucleotide database BLASTn, SKA49 shows nucleotide homology to other members of genus *Carltongylesvirus* viruses. However, the homology was less than 95% to other *Carltongylesvirus* phages therefore it was classified as new species as per ICTV rules and named as *Escherichia* phage SKA49. When analyzed in VIRIDIC software maximum nucleotide homology of SKA49 was observed for *Escherichia* phage vB_EcoM_Bp10 (Bp10, 85.4%) then for *Escherichia* phage Mangalitsa (Mangalitsa, 84.6%), *Escherichia* phage flopper (flopper, 84.5%), *Escherichia* phage vB_EcoP_Bp7 (Bp7, 84.1%), *Escherichia* phage ST32 (ST32, 84%), and *Escherichia* phage phiEcoM-GJ1 (GJ1, 81.5%). All these phages are either members of genus *Carltongylesvirus* or unclassified *Carltongylesvirus* of sub-family *Cleopatravirinae*, in family Chaseviridae (Fig. 3). A cluster table generated by VIRIDIC software indicating SKA49 species and genus placement along with its close homologs is given in supplementary Table S6A.

**Figure 4:**
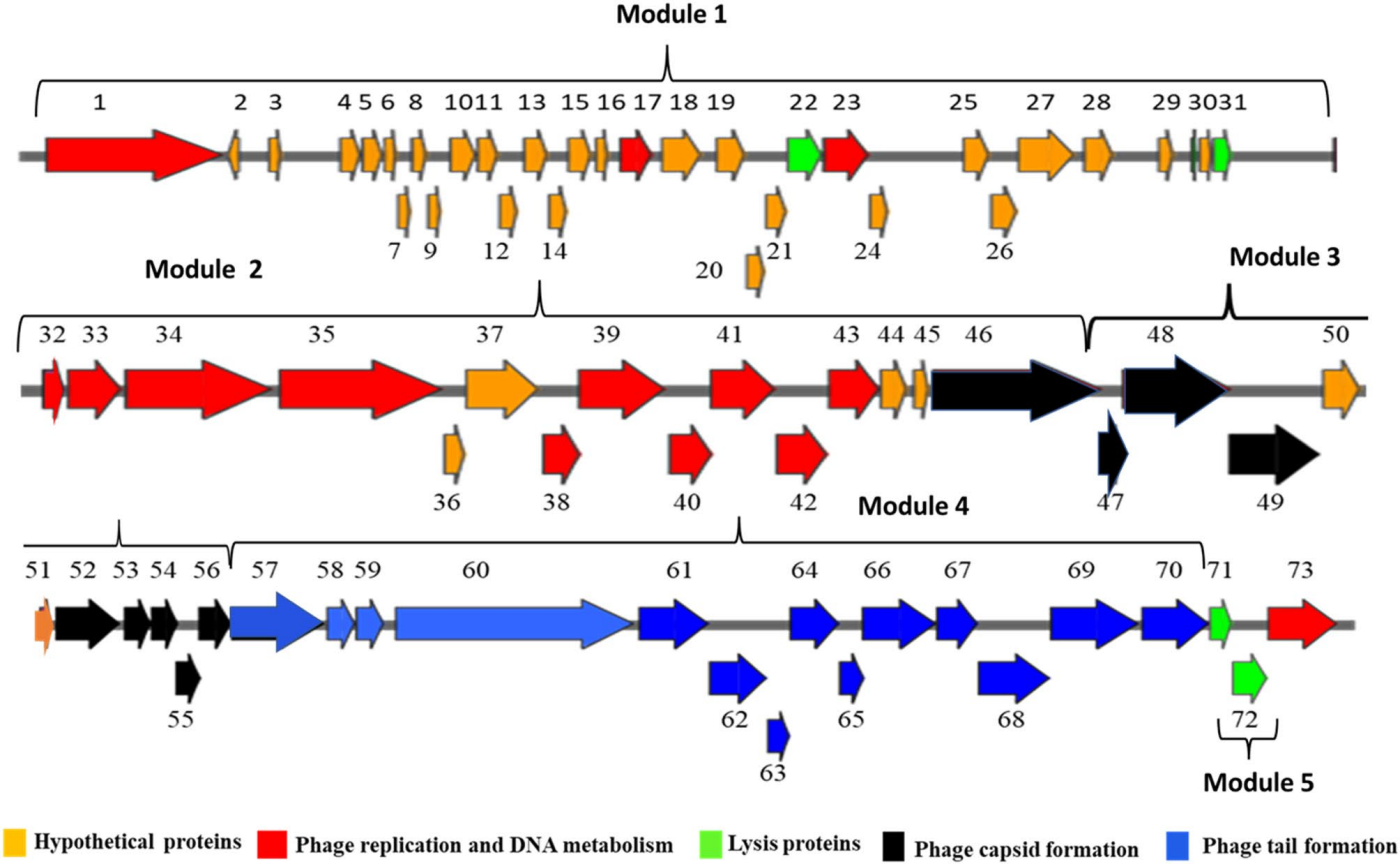
Genome map of *Escherichia* phage SKA49 (51.548 Kb). All ORFs are color coded according to their putative functions. Direction of Arrows indicate replication strand (+ or –). ORFs colored Red are characterized protein, Orange ORFs are hypothetical proteins, Black ORFs represent phage capsid proteins, blue ORFs are involved in tail formation. Lysis cassette is represented with green color. ORF numbers corresponding to their description in Table S7, are given at the top of each ORF.

SKA49 exhibits a modular organization as proteins involved in similar functions were clustered together. A genome map of SKA49 is given in Fig. 4. For ease of description genome is divided into five modules^1–5^. In total SKA49 genome encodes 72 Open Reading Frames (ORFs). Each ORF is labelled according to its description in supplementary Table S7, and it is color coded to indicate putative function. Out of 73 ORFs, 64 ORFs started with ATG start codon, 6 proteins including one hypothetical protein (ORF 8, 29, 35, 49, 63 and 64), started with GTG start codon . Additionally, two proteins including dNMP kinase (ORF 42) and a hypothetical protein (ORF 54), used TTG as start codon. They all specified methionine as start codon. ICTV core gene analysis as well as CoreGenes 5.0 analysis identified 52 ORFs out of 73 as core genes in SKA49 specific for the entire genus of *Carltongylesvirus* when compared to its close homologs in NCBI (supplementary Table S8).

Module 1 included majority ORFs encoding hypothetical proteins with unknown functions (ORFs 2–30, Fig. 4, Table S7). These proteins were genus specific as they exclusively matched genus *Carltongylesvirus* or unclassified *Carltongylesvirus* with no other hits in BLASTn. These proteins are small, ranging from 42 AA (ORF 2, putative size 4.9 kDa) to 224 AA (ORF 26, putative size 25.8 kDa). HHpred software generated no specific hits for these proteins. In addition to these, two proteins involved in initial processing of phage DNA such as ssDNA binding protein (ORF 17) and a putative anti restriction protein (ORF 23) involved in neutralizing host defenses were also present in module 1. Module 2 included 14 ORFs for encoding proteins involved in DNA metabolism, replication, and gene processing (ORFs 32–45).This phage has genome replication autonomy as it encodes for DNA helicase (ORF 34), DNA polymerase (ORF 35), RNase H (ORF 39) and a DNA ligase (ORF 41). In addition, the phage has several ORFs that encode proteins involved in DNA metabolism (ORF 33, 40, 42 and 43). In addition, this module carries 4 hypothetical proteins (ORFs 36,37,44 and 45) with high nucleotide and protein homology with only members of genus *Carltongylesvirus*. Modules 3 and 4 possess genes that code for phage structural proteins. Module 3 represents ORFs involved in capsid formation (ORFs 46–56, Fig. 4, Table S7), and they were highly conserved throughout the genus apart from head tail adaptor protein (ORF 53) that was missing from few phages of genus *Carltongylesvirus.* Module 4 included 14 ORFs involved in tail formation (ORFs 57–70, Fig. 4, Table S7).

**Figure 5:**
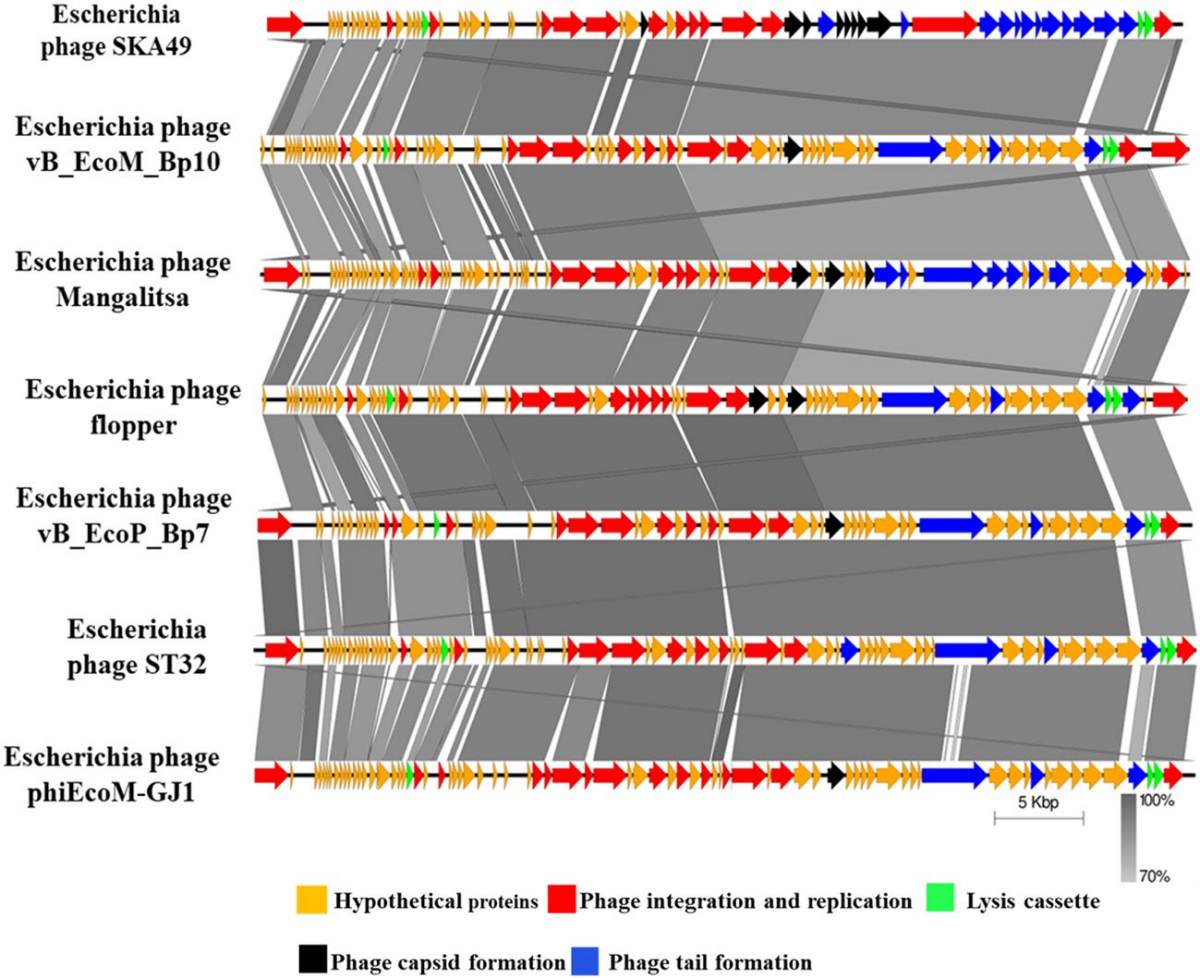
Easyfig homology diagram of SKA49 with close phage relatives in NCBI using BLASTn. Various ORFs are color coded according to their putative function. (Scheme provided at the base of figure). Intensity of shade indicates homology percentage.

Nucleotide sequence of entire SKA49 tail module has highest similarity to phage Mangalitsa (94% coverage and 90% similarity). This module included three distinct sections. Section one included ORFs involved in tail tube formation. The second section included genes involved in base plate formation and tail tube initiation whereas third section included genes involved in tail fiber formation. The first two sections (tail tube and base plate ORFs) were conserved with genus specific homology however the third section tail fiber related proteins were less conserved. ORF 70, a putative tail fiber protein appears to be a chimera between the two phages. Nucleotide sequence of 5’ end of this tail fiber gene (ORF 70) had ~ 70% homology (albeit with low query coverage 65%) with *Escherichia* phage flopper whereas the 236 nucleotides at the 3′ end had high homology (84%) with *Escherichia* phage Mangalitsa. Roughly 400 nucleotide sequence in between these two was unique to SKA49 (supplementary Figure S2). The 5′ end of ORF 70 protein has an R type Pyocin knob domain a putative high molecular weight bacteriocin involved in binding of core LPS of host bacterial strain. This domain was present only in members of genus *Carltongylesvirus*, however maximum homology of SKA49 domain was observed with *Escherichia* phage flopper (78% amino acid homology). The comparison of tail fiber is given in supplementary Figure S2. SKA49 has two putative cell wall hydrolases distributed in two modules of the genome (Fig. 3). A putative L-alanyl-D-glutamate peptidase hydrolase (ORF 22) and a second cell wall hydrolase was present in holin- hydrolase combination (ORF 70 and 71) and a putative spannin protein (ORF 31) (supplementary Table S7). A comparison of SKA49 proteins with its close homologs is given in Fig. 5.

### Genome analysis of *Escherichia* phage SKA64

**Figure 6:**
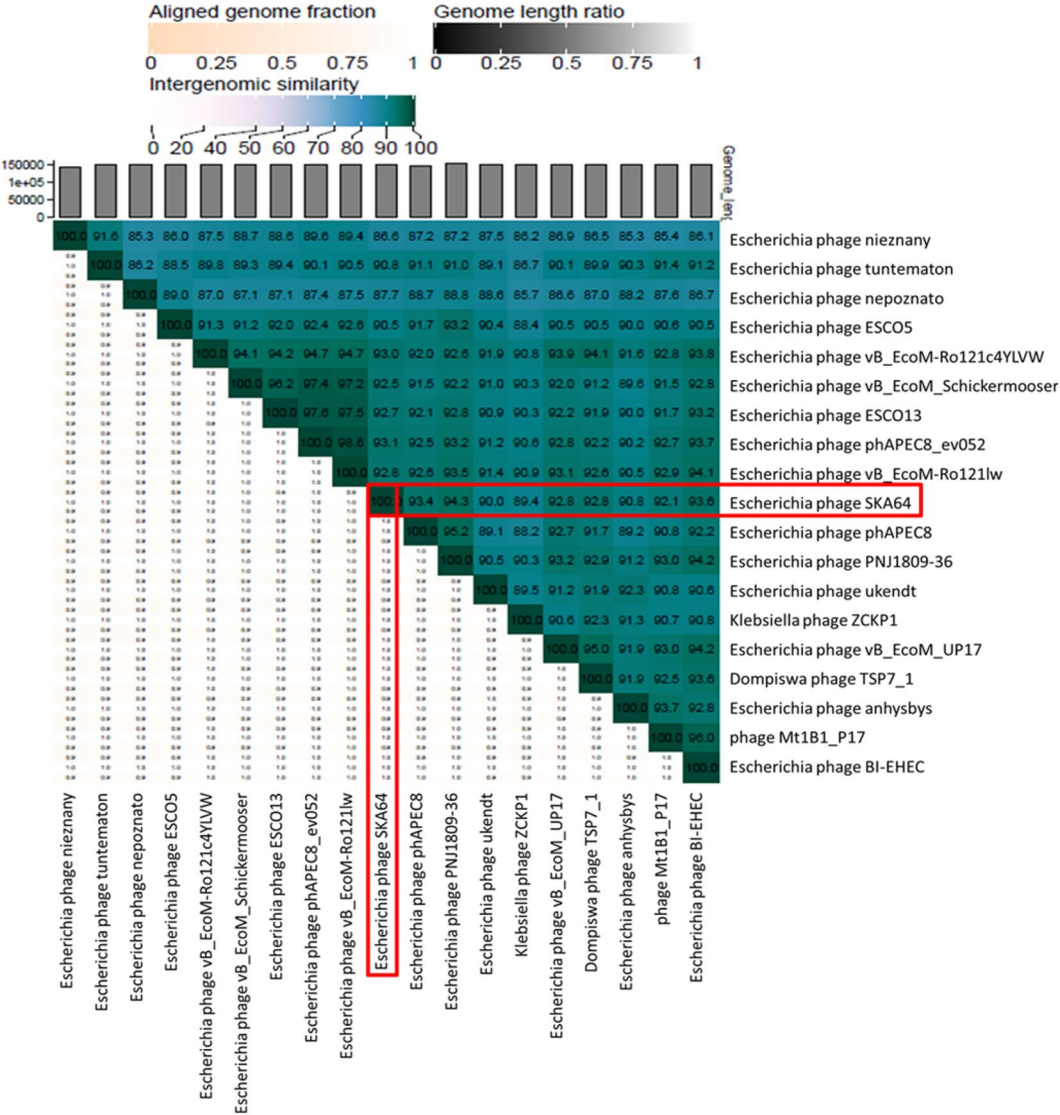
Comparative genome analysis of *Escherichia* phage SKA64 using VIRIDIC software (http://rhea.icbm.uni-oldenburg.de/VIRIDIC/) as per ICTV recommendations. The heatmap generated indicates the relationship of SKA64 with its close homologs in BLASTn and gives an accurate estimation of genomic similarity between phages.

*Escherichia* phage SKA64 is a member of the subfamily *Stephanstirmvirinae* genus *Phapecoctavirus*. It has 152.401 kb genome with 39% GC content. When compared in BLASTn SKA64 was closely related to members of genus *Phapecoctavirus* however this homology was less than 95% (supplementary Table S6B). It showed maximum homology with *Escherichia* phage PNJ1809-36 (94.3%), followed by *Escherichia* phage BI-EHEC (93.6%). A heat map for percentage nucleotide homology between close homologs of SKA64 generated by using VIRIDIC software is given Fig. 6.

**Figure 7:**
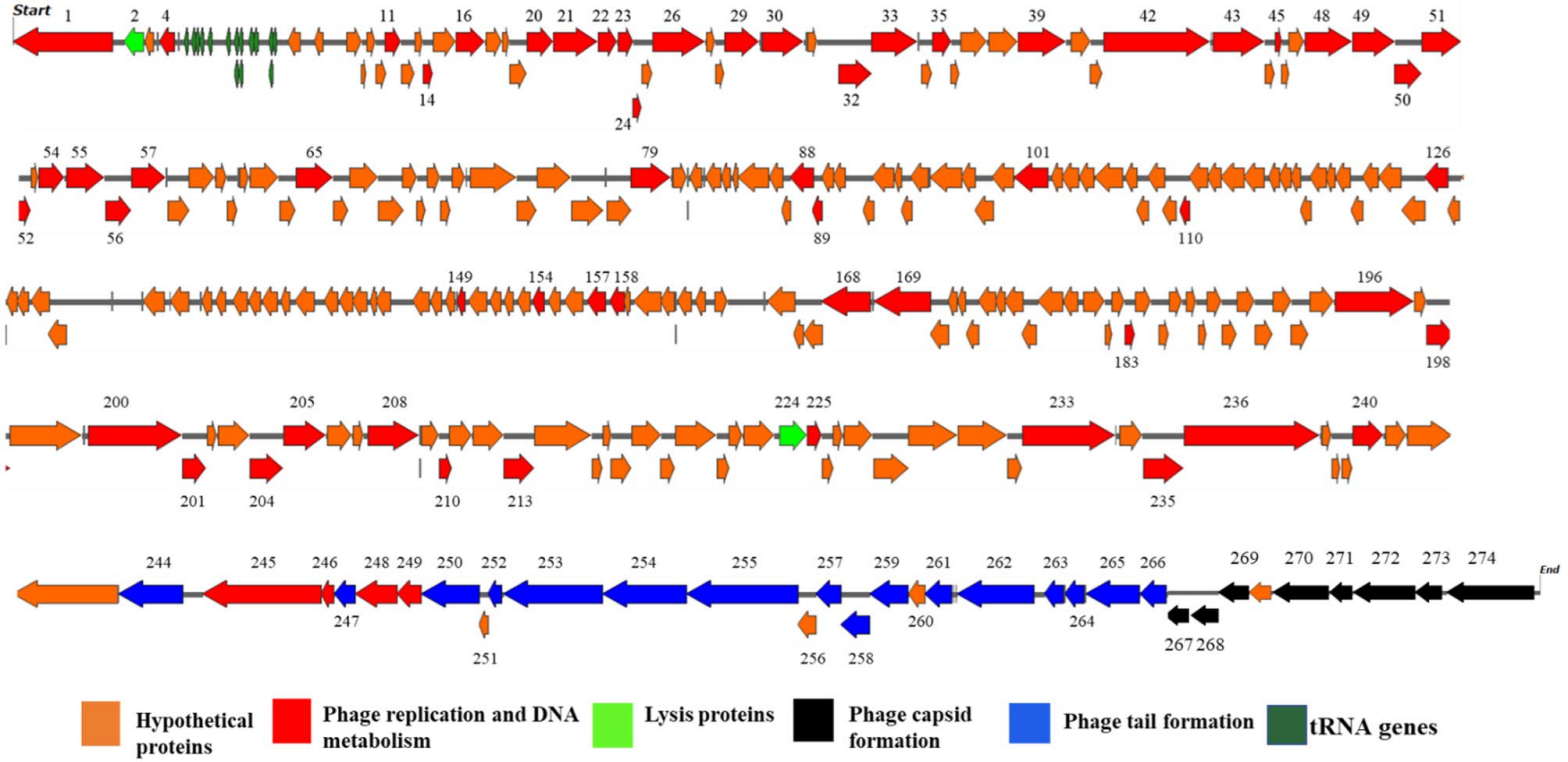
Genome map of *Escherichia* Phage SKA64 (152.401 Kb). All ORFs are color coded according to their putative functions. Direction of Arrows indicate replication strand (+ or –). ORFs colored Red are characterized protein, Orange ORFs are hypothetical proteins, Black ORFs represent phage capsid proteins, blue ORFs are involved in tail formation. Lysis cassette is represented with green color. tRNA genes are labelled olive-green color. ORF numbers corresponding to their description in Table S9, are given at the top of those ORFs whose putative function is known.

The genome map of SKA64 is given in Fig. 7.The genome has 275 ORFs that are color coded according to their putative function. Only those ORFs are labelled by a number whose function is known. Out of total 275 ORFs vast majority are hypothetical proteins (188 ORFs) having BLASTn homology with phage proteins of unknown functions (color orange). Out of remaining 87 ORFs having BLASTn homology with characterized proteins,16 ORFs were involved in tail formation (color blue) and 8 ORFs in capsid formation (color black) whereas others were conserved proteins involved in DNA metabolism and replication (color red). SKA64 has a large pool of tRNA genes including two for Methionine, 3 for Selenocysteine and one each for Isoleucine, Proline, Glutamine, Glycine, Threonine, Asparagine, Tyrosine, Serine, and Arginine. These tRNA genes are clustered together after large subunit of phage terminase. The presence of these tRNAs indicate codon usage autonomy of SKA64 (color olive green).

**Figure 8:**
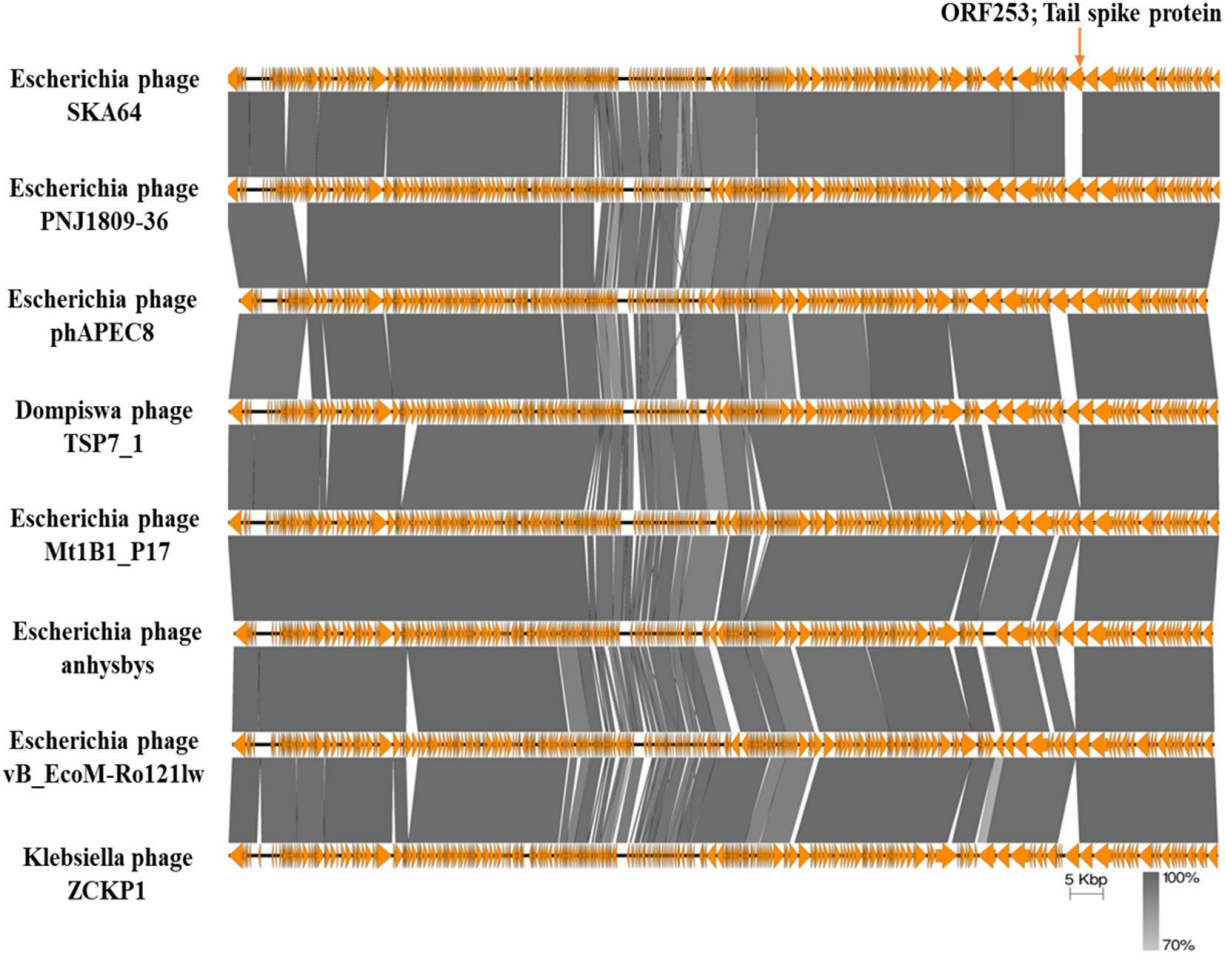
Easyfig homology diagram of SKA64 with close phage relatives in NCBI using BLASTn.

All ORFs involved in tail formation (colored blue, Fig. 7) are conserved throughout the genus except for SKA64 tail spike protein (ORF 253, 2550 bp, 849AA). This protein has no nucleotide or protein homology with any member of genus *Phapecoctavirus* or even sub-family *Stephanstirmvirinae*. The closest homolog of this tail fiber protein is *Escherichia* phage Pisces and Penshu1, both from genus *Kayfunavirus* from another family. SKA64 has a non-contractile tail sheath like other members of this genus. The nucleotide sequence of capsid genes is conserved in the genus. SKA64 has a large pool of genes involved in replication, transcription, nucleotide metabolism, and DNA repair. An Easyfig diagram depicting comparative analysis of SKA64 with its close homologs in BLASTn is given in Fig. 8. CoreGenes 5.0 analysis indicated 215 ORFs as core genes of the genus out of 275 ORFs. This further strengthens the placement of SKA64 in genus *Phapecoctavirus* (supplementary file 2). The genome map has labels for all these genes that correspond to their description in supplementary Table S8.

SKA64 utilizes a spore cortex-lytic enzyme of hydrolase 2 superfamily and is predicted by HHpred to be a lytic transglycosylase (https://toolkit.tuebingen.mpg.de/tools / hhpred). SKA64 has a recombinase family protein (ORF 101, supp. Table S8) that is in nature a resolvase responsible for keeping phage DNA concatemers as single genome. SKA64 had homology with only phage genomes and no prophage matches were found which indicates it does not integrate in host genome.

### Suitability of SKA49 and SKA64 as probable therapeutic/biocontrol agents

One of the important characteristics required for any phage for putative therapeutic/ biocontrol use is that it must be exclusively lytic, possessing no integration / recombination genes in its genome. Moreover, it must not have any virulence genes or antimicrobial resistance markers. To test the suitability of SKA49 and SKA64 as probable candidates for therapy/ control of bacteria they were subjected to Phage Leads software analysis. The absence of integration and recombination genes was indicated by Phage Leads software predicted that both phages may be following lytic pathway. Both SKA49 and SKA64 had no virulence and antimicrobial resistance genes as indicated by Phage Leads software analysis.

### Phylogenetic analysis of SKA49 and SKA64

**Figure 9:**
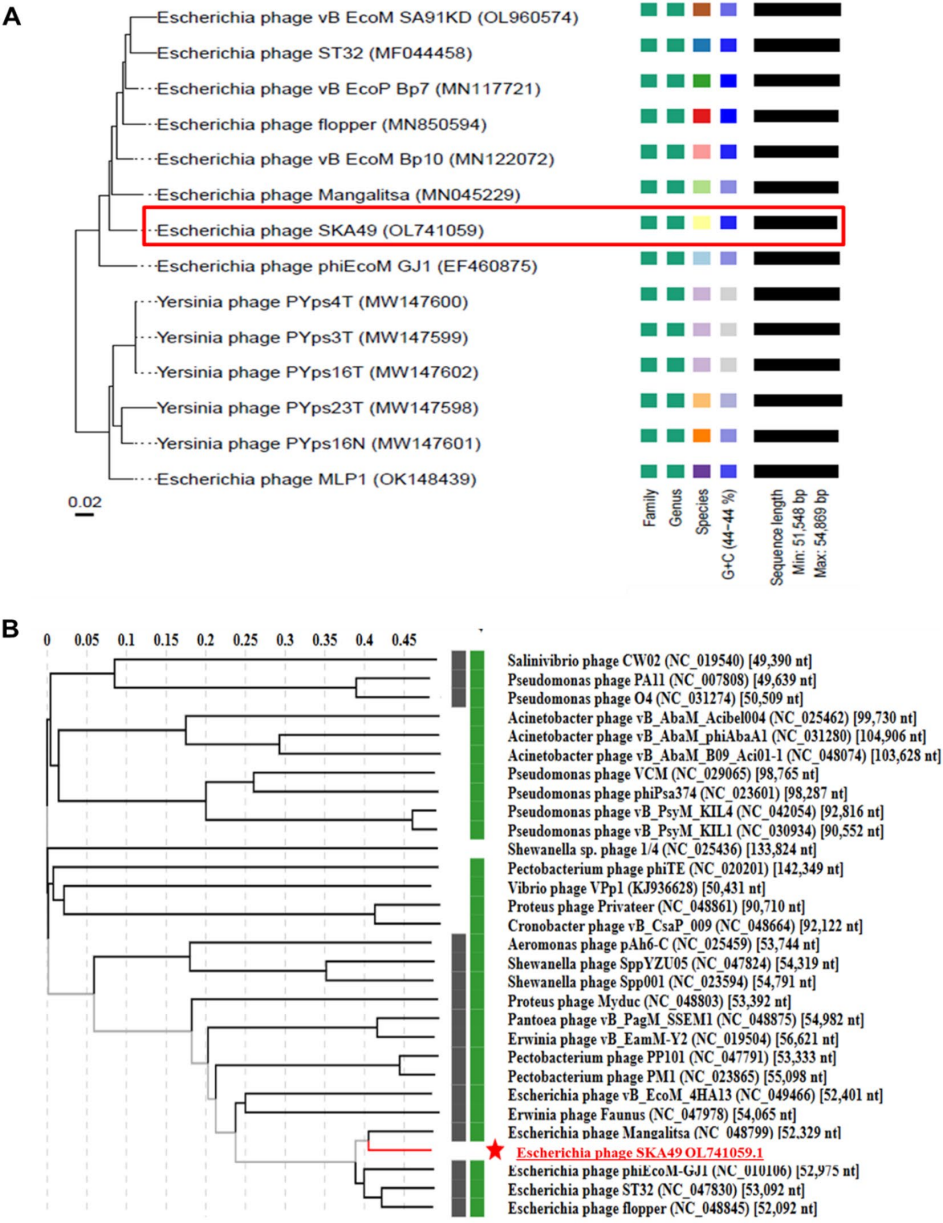

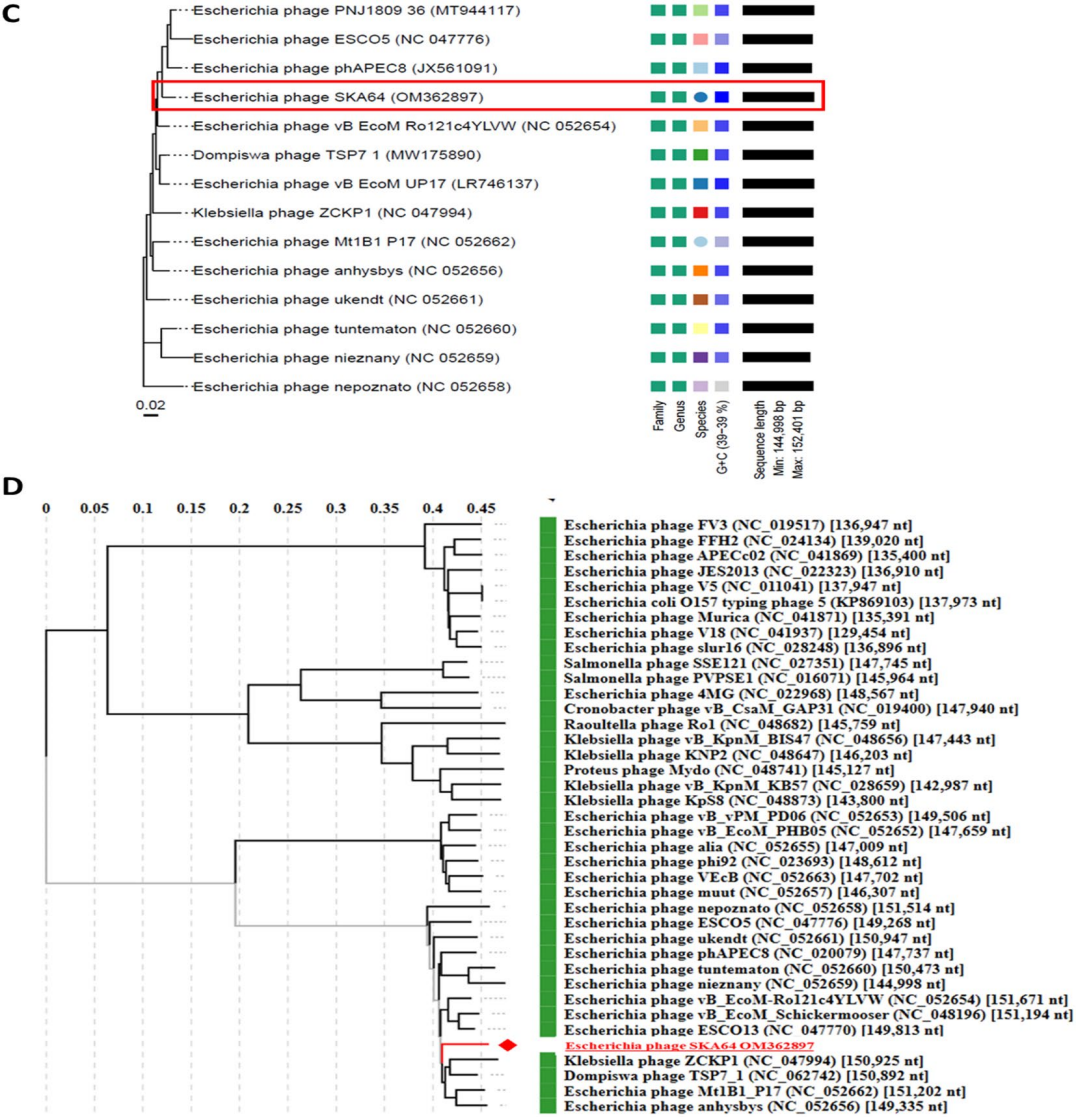
(**A** and** B**) Phylogenetic position of *Escherichia* phage SKA49. (**A**) Nucleotide based GDBP tree provides an estimate of phage relationship with close homologs in BLAST n using VICTOR. Analysis identified one Family, one genus, and 12 species clusters respectively, identifying SKA49 as new species. (**B**) A proteome tree made by ViPTree software that uses genome wide similarities calculated by tBLASTx. Genomes for comparison were selected according to SG scores of 30 sequences in VIP tree ranging from 1 to 0.02 (supplementary Table S10B) (**C** and **D**) Phylogenetic position of *Escherichia* phage SKA64. (**C**) Nucleotide based GDBP tree provides an estimate of phage relationship with close homologs in BLAST n using VICTOR. Analysis identified one Family, one genus, and 14 species clusters respectively, identifying SKA64 as new species. (**D**) A proteome tree made by VIP Tree software that uses genome wide similarities calculated by tBLASTx using Similarity score generated by software . Both trees place SKA64 in the same genus. Genomes for comparison were selected according to SG scores of 39 sequences in VIP tree ranging from 1 to 0.07 (supplementary Table S10B).

Genome-BLAST Distance phylogenomic (GDBP) tree was produced using close homologs of SKA49 and SKA64. The comparative analysis and accession numbers of these taxa for both phages are given in supplementary Tables S9A and S9B respectively. The phylogenomic GBDP tree inferred using the formulas D0 for SKA49 is given in Fig. 9A. It yielded average support of 7%. The branch lengths of the resulting VICTOR trees are scaled in terms of the respective distance formula used. For SKA49 The OPTSIL clustering yielded twelve species clusters. All except one (OK148439) *E. coli* phages clustered together, and all *Yersinia* phages clustered together. OK148439 appeared as a basal group to *Yersinia* phages. The SKA49 phage (OL741059) appeared next to the basal group (EF460875) of the *E. coli* phage cluster. This analysis identified 12 species clusters and SKA49 was closest to *Escherichia* phage Mangalitsa and GJ1. To generate proteomic tree for SKA49 ViPTree software was used to compare genome wide sequence similarities using tBLASTx. All phages having genome similarity score between 1- 0.02 for SKA49 (30 phages) were included in phylogenetic analysis (supplementary Table S10A). It further validated placement of SKA49 in genus *Carltongylesvirus* closest to GJ1 and Mangalitsa. This tree placed *Escherichia* phage Mangalitsa, *Escherichia* phage ST32 and *Escherichia* phage flopper in one cluster recognizing SKA49 as a separate species in the same genus. Tree represents SKA49 relationship with close homologs and outliers (Fig. 9B).

For SKA64 the GDBP tree was inferred using D0 formula yielding an average support of 2%. OPTSIL clustering produced fourteen species clusters for SKA64 identifying it as a separate species in the genus (Fig. 9C). When nucleotide similarities were compared SKA64 has closest homology with *Escherichia* phage phAPEC8. SKA64 proteome tree was generated using 39 phages showing genome similarity range of 1- 0.07 in a genome similarity table produced by ViPTree software (supplementary Table S10B). According to proteomic tree SKA64 was more closely related to *Klebsiella* phage ZCKP1 and *Escherichia* Phage ESCO13. Both analysis place SKA64 in genus *Phapecoctavirus* as a new species (Fig. 9D).

**Figure 10:**
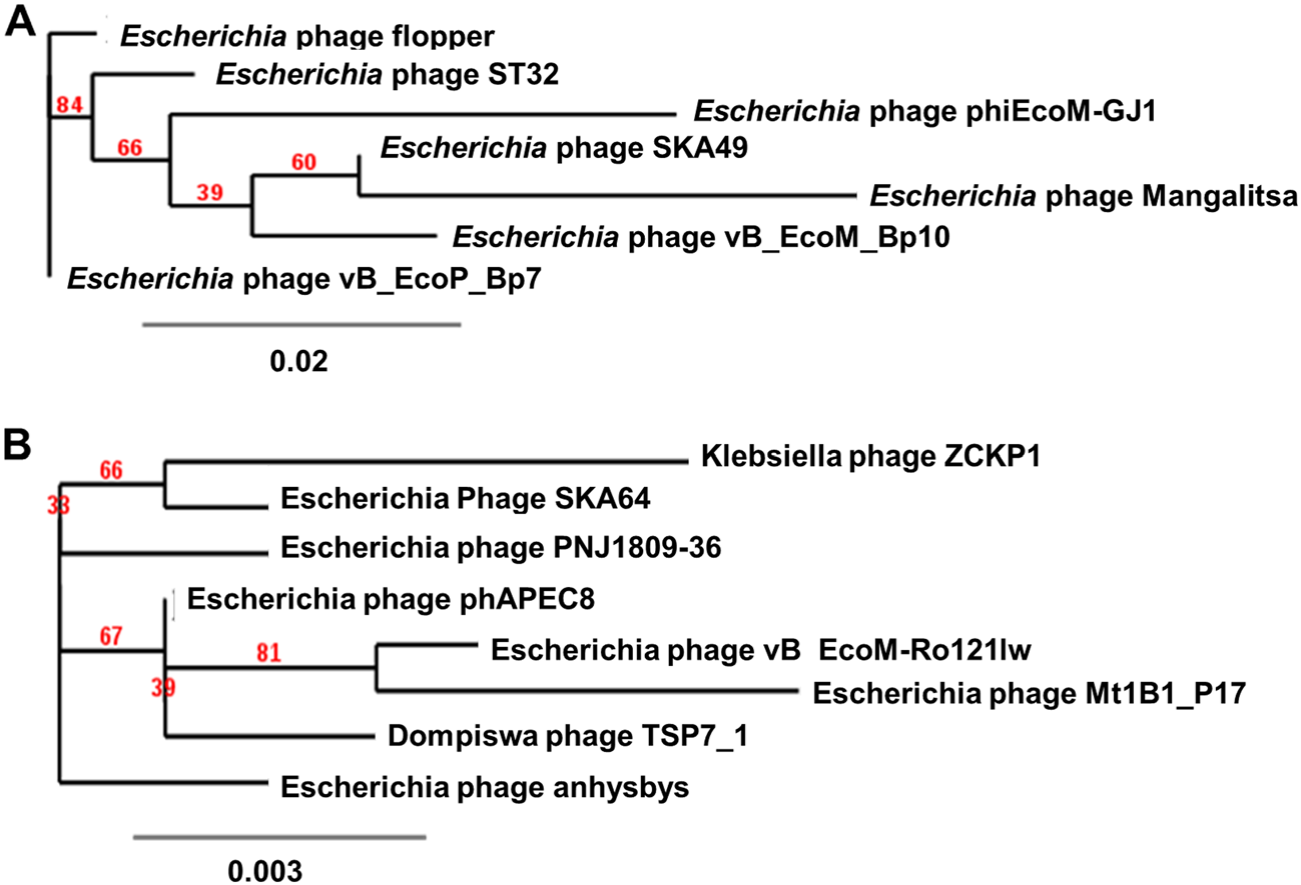
(**A** and **B**) Maximum Likelihood Tree (MLT) using orthologs from CoreGenes analysis. (**A**) MLT for SKA49 using amino acid sequence of Major capsid protein of close homologs. (**B**) MLT generated by amino acid sequence of Tail fiber protein from close homologs.

Furthermore, we also generated a maximum likelihood tree using amino acid sequence of one ortholog identified in CoreGenes analysis for both phages. This tree was produced using TreeDyn software. For SKA49 Major Capsid gene was used. Software produced and alignment of 336 amino acids. The best fit model on the data was found to be WAG + I. Tree produced is given in Fig. 10A. It placed SKA49 closest to *Escherichia* phage Mangalitsa, further strengthening our analysis.

For SKA64 Tail fiber protein was selected as the ortholog and a maximum likelihood tree was produced using an alignment of 959 amino acids. The tree places SKA64 closest to *Klebsiella* phage ZCKP1 like ViPTree analysis Fig. 10B.

## Discussion

This study was designed to find lytic bacteriophages against field isolates of APEC ,having suitable characteristics for their putative biocontrol / therapy application against MDR poultry strains of zoonotic potential circulating in capitol territory Pakistan. There were similarities in phylogroup distribution, virulence profiles and resistance patterns in *E. coli* strains isolated from poultry and human ICU patients. It is established that PCR based phylotyping has 80–95% concordance with MLST analysis, providing an effective, cheaper, and rapid tool to understand the genetic diversity of strains of *E. coli*^2,3^. Strains from different phylogroups vary in their genome size, gene content, pathogenicity, ecological niche, and life history characteristics^25^. Phylogenetic analyses of *E. coli* strains from different sources indicate that virulent extraintestinal *E. coli* strains belong mainly to group B2 and D. In contrast, most of the commensal strains are associated with group A or group B1^4,5^. Our results indicated that phylogroup D was common to both human and poultry isolates. Moreover, most Extraintestinal pathogenic *E. coli* (ExPEC) causing UTIs in humans belong to phylogroup D^26^. Common virulence determinants of APEC, UPEC and NMEC include *traT, iroN, vat, papC, kpsMTII, papEF, ibeA, pic, iss, ompT, cvaC, and fimH* etc.^27^. Both poultry and human *E coli* isolated in our study possess *Aggr*, *ecpA*, *vat*, *traT, papC, ompT and fimH as common virulence markers* which emphasize their zoonotic potential*.* One such isolate, QZJM25 from phylogroup D having these virulence markers and an extensive drug resistance profile was selected for isolation of *E coli* specific bacteriophages.

As per ICTV recommendations any new bacteriophage can be classified in to one of the existing genera if it has more than 70% nucleotide homology or 40% of its proteins have ≥ 70% homology with members of that genus^28^. Since SKA49 has between 81.5%- 85.7% nucleotide homology with members of *Carltongylesvirus* as well as majority proteins have more than 80% homology to members of this genus it is included in un-classified *Carltongylesvirus* division. Whereas SKA64 belongs to genus *Phapecoctavirus* in subfamily *Stephanstirmvirinae.*

To fulfil ICTV requirements a heat map was generated using VIRIDIC software for genome wide comparison of both phages with their close homologs in respective genera*.* This heat map identified both SKA49 and SKA64 as new species. SKA49 proteins have high homology with three phages Mangalitsa (27%), ST32 (26%) and GJ1 (15.1%), however only ST32 is physically characterized and for remaining two only genomes have been reported^29,30^. Likewise, for SKA64 only genome sequence of few closely related homologs have been reported but none of them is fully characterized^31,32^.

Several studies have reported characterization and use of bacteriophages against APEC strains of poultry origin. A study performed by Kazibwe et al. isolated and characterized seven lytic bacteriophages against APEC strains isolated from broilers. These phages had moderate host ranges and were not stable at temperatures above 60 °C^19^. Another study conducted by Tang et al., also report similar findings and good potential of lytic phage CE1 to act as surface disinfectant as well as its therapeutic potential to control APEC challenge in vivo in 1-day old broiler chicken^21^.

Utility of any phage in biocontrol / therapy depends on a list of characteristics defined in detail by several publications^22,33–35^ First requirement is that phage should be exclusively lytic to control the target bacteria to sub-infectious levels^36^. Both SKA49 and SKA64 are predicted to be lytic as their genomes do not have any ORFs involved in integration and recombination as indicated by PhageLeads software analysis. Both phages were also free of any virulence, toxin, and antimicrobial resistance genes. SKA64 possesses a recombinase family protein (ORF101) that is resolvase/ invertase probably for keeping the phage DNA concatemers in single molecules. Same is true for other phages of their respective genera.

Lysis potential of both phages cannot be compared with other phages of their respective genera as none of them is tested for their ability to control bacterial growth. However, both SKA49 and SKA64 were able to reduce the bacterial growth keeping it significantly lower than the control for 18 h. They were not tested beyond 18 h. Both phages were tested for bacterial growth reduction at two different MOIs and the difference between their efficacy in controlling bacterial strain was statistically non-significant. Generally higher MOIs are more efficacious than low MOI^37^ however, the assay was carried out with slight modification where bacteria and phage were mixed in 1;1 and 1;100 ratio in 1 ml and then diluted to final volume of 40 ml after sufficient phage absorption. This may allow better contact of phage and bacteria to cause efficient infection. Infection of phage to any host is presumed to be controlled by phage to bacterium ratio however MOI does not give account of the total infected bacteria^38^. Similar findings were reported by Liu et al. where MOI value had no significant effect on bacterial infection and appearance of resistance mutations till 80 generations^39^.

Another desired characteristic of phages isolated for biocontrol and therapeutic applications is their stability at normal temperatures for storage. Temperature tolerance analysis indicated that both phages were stable at 37 °C. The same was true for a close homolog of SKA49, *Escherichia* phage ST32 who was stable between 20 and 37 °C^30^. SKA49 was not tested at 20 °C. However, another member of the same genus, Bp10 was reported to be stable at 30 °C- 60 °C temperature^40^. SKA49 was stable at pH 7 and was able to grow at pH5 ad 9 however with a significant drop in titer. Bp10 its close homolog however was stable at all three pH values with no drop in titer. pH stability was not reported for ST32. SKA64 was stable at only pH7 and did not produce any plaques at other pH values. Inability of both phages to stay stable at wide range of pH values may restrict their application in therapeutic applications in vivo to only nasopharyngeal route. Since both phages do not tolerate pH below 5, it may restrict their ability to pass through stomach (pH 2–3.5). However, APEC strains are usually associated with severe respiratory problems which later lead to bacteremia and colibacillosis. Application of phages through nasopharyngeal route/ intra-muscular route may preserve their efficacy. SKA49 and SKA64 may also be employed for biocontrol applications on poultry meat surface . It requires a pH range between 6.5 and 7.3 which is well tolerated by both phages^41^.

SKA49 was effective against 48% of APEC strains tested, whereas SKA64 was effective against only 14% poultry strains tested. One major reason could be that phages were tested against field isolates rather than standardized reference strains and they were not evaluated for presence of any inducible prophages. These prophages if present can make bacterial strain immune to superinfection with another phage^42^. Narrow host range is a major limitation for use of phages in therapy however, such phages can be used in combination with other broad host range phages and antimicrobials to get maximum benefit of their lysis potential^43^. SKA64 tail spike protein is completely unique for members of *Phapecoctavirus* genus. In a recent study Lone Brøndsted has established that tail spike protein of phages interact with bacterial receptors and subtypes of tail spike protein may shed light on the selected host range of phage. It is likely that SKA64 narrow host range is attributed to this recombination event that led to unique tail spike protein^44^.

The burst size of SKA49 was low (56 pfu/cell), this observation is consistent with the fact that ST32 had a very low burst size at 37 °C but improved when phage grown at 20 °C. Same was true for Bp10 another close homolog of SKA49 whose burst size was 56 virions/cell. Therefore, low virion counts of SKA49 at 37 °C is expected as it belongs to cold range phages^45^. Similar comparisons for SKA64 were not possible as none of the close homologs are characterized for their stability, only genomes of few have been reported.

To deploy a phage in therapeutics its genome must be tested for presence of any virulence genes / recombinases and integrases^34^. For this reason, the genome of a therapeutic phage must be characterized. The SKA49 phage genome has 44% and SKA64 has 39% GC content which is the same as reported for other members of their respective genera (Supplementary Tables S10A and S10B). SKA49 has only one tRNA gene (tRNA_Arg_) for coding arginine (from 13,796 to 13,887, 92 bp) like ST32 , Flopper^46^and GJ1, however it has no intron in this gene unlike GJ1^47^. Unlike SKA49 , SKA64 possesses 15 tRNA genes. Phages carry tRNA genes to compensate for genomic differences between them and their host. Usually, these tRNA are used for codons highly preferred by phages in contrast to their host^47–49^. It indicates that SKA64 has more translational autonomy in comparison to SKA49. These tRNA genes were conserved in the entire *Phapecoctavirus* genus.

The tail fiber proteins of phages are usually highly mosaic in nature. An excellent example of such mosaic arrangement is ORF 70 , the tail fiber protein of SKA49 where 5’ and 3’ ends of the ORF seems to be contributed by two different phages (phage Flopper and Mangalitsa respectively). Downstream to the N terminus of this protein a pyocin knob domain has significant protein homology (78%) to phage Flopper only. This pyocin domain is unique to SKA49 and Flopper as no significant BLASTp homology with other phages of the same genus was identified (supplementary Figure S2). These R1 and R2 type of pyocin are powerful bacteriocins used by *pseudomonas* sp. for eliminating competition^50,51^. Tail spike protein of SKA64 was unique as it has no protein as well as nucleotide homology to any member of *Phapecoctavirus* genus, rather it resembled (53%QC with ¬ 79% homology) to phages *Escherichia* phage Pisces and Penshu1, from another genus (Kayfunavirus) of a different family. It agreed with the data presented by Haggard in 1992 indicating that phages can exchange tail fiber genes even though they are not related. It may result in their host range expansion or contraction, and it may allow them to bind alternative receptors on the same host^52^.

SKA49 has two distinct types of endolysins. One of these endolysins is present in the first genome module (ORF22 Fig. 4) and this hydrolase is a D-Ala-D-Gln carboxypeptidase that can lyse D-glutamate and L-alanine residues in glycoprotein portions of bacterial cell wall from within the cell. Such endolysins are part of M15 superfamily of proteins first identified in Listeria monocytogenes (Ply118)^53,54^. Interestingly the N’ terminus of this hydrolase till 80AA was conserved in the genus however C’ terminus from 80-138AA matches only SA91KD and ST32 phage and for all others it was partially similar. GARD analysis (Genetic Algorithm for Recombination Detection; http://www.datamonkey.org/GARD) showed that this hydrolase possesses a recombination hotspot between amino acid 81 and 82 and places endolysin of SKA49, ST32 and SA91KD in one lineage whereas others diverted (supplementary Figures S3A and S3B). similar phenomenon is reported by Oechslin^55^. The second endolysin of SKA49 is a putative SAR endolysin whose ORF clustered together with a pin-holin and a distant spannin (ORF 71,72,& 31, Fig. 4). It belongs to a new class of endolysins called “true lysozymes”^51^. These lysozymes possess an SAR (Signal-Anchor-Release) sequence (as analyzed by HHpred software) that exports this lysozyme to the periplasm in holin independent manner in an inactivated form to prevents premature cell lysis^56^. These enzymes may be able to degrade bacterial cell wall independent of holin by changing protonmotive potential of the outer membrane^57,58^. SKA64 has only one endolysin (ORF 224) that belongs to hydrolase 2 superfamily and is a transglycosylase enzyme^59^.

This detailed characterization of SKA49 and SKA64 will not only add to the existing knowledge about their respective genera it will also pave way for their probable use in therapeutic/ biocontrol applications after in vitro and in vivo evaluations.

## Methods

### Isolation and confirmation of bacterial strains

*Escherichia coli* strains used in this study were isolated and characterized in Molecular Virology labs (MVL), CUI to identify endemic *Escherichia* coli species in poultry showing signs and symptoms of *E. coli* infection. All methods used in this manuscript have been adapted from established protocols are appropriately cited. For isolation standard protocols as illustrated by^60,61^ were used. Briefly, sixty random poultry samples including liver, intestine, caecum, and spleen were collected from different poultry farms in federal area, Pakistan in sterile zip lock bags and stored on ice during transportation.

Thirty patient urine samples with suspected urinary tract infection from ICU patients of three major hospitals in federal area (Rawalpindi and Islamabad), Pakistan were also included in this study. These UTI samples were collected in a previous study at MVL CUI following appropriate guidelines (Helsinki Declaration: https://www.wma.net/policies-post/wma-declaration-of-helsinki-ethical-principles-for-medical-research-involving-human-subjects/) and plated on to MacConkey agar plates on-site in hospital pathology lab by hospital staff after informed patient consent on a pre-approved proforma, and placed in incubator at 37˚C. No human tissue samples were included in the current phage study or sampling was carried out directly from individuals for this study therefore no regulatory approvals were obtained. Poultry samples were processed at MVL CUI. For processing 20 g of each poultry tissue were pooled together in a sterile disposable petri plate and washed with PBS twice. Later this pooled sample was macerated in PBS to fine paste using sterile disposable scalpel blade in a glass Petri plate. Macerated tissue sample was enriched in 10 ml buffered peptone water over night at 37 °C^62^. After 24 h a loop full of enriched medium was streaked on MacConkey’s agar (Oxoid; CM0007B) plates and incubated at 37 °C for 18–24 h. Appearance of lactose fermenting pink colonies on MacConkey’s agar indicated presence of *E. coli*. Both poultry and human suspected *E .coli* strains were further confirmed by biochemical analysis using API 20E kit strips (bioMérieux). Those strains having appropriate biochemical/ numeric profile of API kit as per manufacturer’s instructions (Biomerieux) were subjected to molecular confirmation by amplification of 271 bp of *E coli* housekeeping gene β-glucuronidase (*uid*A)^63^ using primers F 5′- TGT TGA CTG GCA GGT GGT A-3 and R 5-TAA AGT AGA ACG GTT TGT GG-3′. This identified 21 poultry and 15 human urine strains as *E. coli* (Supplementary Table S1). All these strains were used for phage host range identification in further experiments (Table1).

### *E. coli* phylotyping

All *E. coli* isolates were classified in to appropriate phylogroups using standard quadruplex scheme given by Clermont and colleagues 2012^3^. Briefly the genomic DNA of all *E. coli* isolates was extracted using PCI (Phenol, Chloroform, Isoamyl alcohol) method^64^ and subjected to PCR identification for presence or absence of four genetic markers *arpA, chuA, yjaA, TspE4.C2* using oligonucleotide primers given in^3^. After gel electrophoresis the isolates were either immediately categorized into a phylogroup based on quadruplex genotype or further confirmed using group specific PCR. Phylogroups were assigned based on the schematic presentation as given by Clermont and colleagues in 2012. Phylogroup of all isolates is given in supplementary Table S1.

### *E. coli* pathotyping

Presence of virulence genes for surface adhesions *pap C, fimH, Aggr, sfa/fogDE* , biofilm facilitator *ecpA*, serum tolerance factor *tra T*, Outer membrane proteases *OmpT* and toxins *Stx1, 2* and *Vat* were tested by PCR amplification of respective genes as described previously^65–70^. The complete list of primers used for 10 virulence factors tested in this study is given in supplementary Table S4.

### Antibiotic sensitivity testing of *E. coli* isolates

Antibiotic sensitivity of all isolates was tested by Kirby-Bauer disc diffusion method. phenotype and genotype of all confirmed *E. coli* isolates was tested against 27 antibiotics commonly employed for treatment of *E. coli* in humans and poultry. Antibiotic discs with standard recommended concentration were used for sensitivity testing (Oxoid) (Table S2 & S3). In brief each isolate at 0.5 OD adjusted at McFarland scale was inoculated on Mueller Hinton Agar (MHA) plates using cotton swabs and left to dry for 30 min at room temperature. Antibiotic discs were applied on each plate using sterile forceps and incubated at 37 °C overnight. Isolates were categorized as sensitive, resistant, or intermediate by measuring the zone of inhibition around each disc according to ‘Performance Standards for Antimicrobial Disk Susceptibility Tests^71^. Any isolate resistant to at least one drug in at least 3 antimicrobial classes was categorized as Multiple Drud Resistant (MDR) isolate^72^. Details are given in supplementary Tables S2 and S3. MAR index was calculated according to standard definition given by Krumperman,^73^. One strain QZJM25 was resistant to 20 antibiotics out of 27 tested. Moreover, it also possessed *bla*_TEM-1_ gene for beta lactamase resistance. Therefore, it was further confirmed by PCR amplification of 16srRNA gene (Macrogen, Seoul, Korea) (GenBank Acc No, OK086691). This strain was used for bacteriophage isolation and characterization. The resistance profile of QZJM25 is given in Supplementary Table S3.

### Bacteriophage isolation

One gram tissue sample of intestine, caecum, and stomach from poultry samples previously tested positive for *E. coli* were pooled and triturated in 10 ml of buffered peptone water and incubated at 37 °C for 24 h without shaking. Next day the samples were centrifuged at 8000 rpm for 10 min (Centrifuge, Hermle, Siemensstr. 25, D-78564 Wehingen, Germany ZK 496) and supernatant was filtered through 0.22 µm syringe filters (CNW Technologies, Düsseldorf, Germany). One milliliter of this supernatant was mixed with 2 ml of QZJM25 overnight culture supplemented with 10 mM CaCl_2_ and incubated at 37 °C for 24 h. Next day the mixture was again centrifuged at 8000 rpm for 20 min to remove bacterial cells and supernatant was filtered through 0.22 µm syringe filter. This was crude phage lysate which was stored at 4 °C and later tested by agar overlay spot method as described by^74^. Briefly, 250 µl of QZJM25 overnight culture was mixed with 2.5 ml of soft agar (Lauria-Bertani (LB)-agar 0.5% w/v) and poured on to the LB agar plates. Plates were solidified for 15 min and then 10 µl drops of crude phage lysate were spotted on the plate. Once the drops were dry the plates were inverted and incubated at 37 °C for 24 h. All samples that produced clear zones of phage lysis on bacterial lawn were selected for further purification.

#### Purification of phages

Usually, the crude phage lysate obtained after phage isolation procedure is a mixture of phages. To purify/ isolate a single phage with good lysis potential the crude phage lysate was subjected to single plaque purification method as describe by^75^. Briefly, 250 µl of QZJM25 overnight culture and 25 µl of phage lysate were mixed in 2.5 ml soft agar in Kimax glass tubes (Merck; Cat # Z255122) for phage titration and then poured on to solidified LB agar plates (Agar overlay method). One isolated plaque was extracted from the plate and suspended in 1 ml phosphate buffered saline (PBS) pH 7.2 and gently rocked for 1 h. Later the mixture was centrifuged at 8000 rpm, for 10 min and filtered through 0.22 µm syringe filters. The entire procedure was repeated twice to obtain pure phage cultures and final plaques extracted were saved at 4 °C. Phages was named *Escherichia* phage SKA49 and *Escherichia* phage SKA64 as per ICTV rules after genome analysis^28^.

#### Large scale amplification of phages

One ml phage lysate obtained after purification was further amplified on a large scale. Briefly an overnight culture of QZJM25 was diluted in 1 L of LB liquid broth (1:100 dilution) and allowed to grow at 37 °C with shaking until culture reaches log phase. At log phase (OD600 0.55) phage lysate at MOI1 was mixed with culture and allowed to grow overnight at 37 °C. Next day culture was centrifuged at 8000 rpm (Centrifuge, Hermle, Siemensstr. 25, D-78564Wehingen, Germany ZK 496), for 20 min to remove bacterial cells. Phage was concentrated using 10%, w/v Polyethylene Glycol 8000 (PEG). PEG (10%) was dissolved in the supernatant obtained after large scale amplification and left at 4 °C overnight for precipitation. Next day the phage-PEG mixture was centrifuged at 10,000 rpm, at 4 °C for 45 min. The precipitate obtained after centrifugation was dissolved in 2 ml PBS (pH 7.2), filtered, and stored at 4 °C until further use^76^.

### Phage stability testing

Both phages were tested for their pH and temperature stability as per standard protocols with few modifications^77^. To test temperature stability a fixed number of phage particles (2.5 × 10^9^ pfu/ ml for SKA49 and 3 × 10^8^ pfu/ml for SKA64) were suspended in 1 ml PBS and incubated at 37 °C, 60 °C, and 80 °C for 1 h. After temperature incubation the phage was titrated, and its viability was checked. To determine pH stability the same number of phage particles (2.5 × 10^9^ pfu/ml and 3 × 10^6^ respectively), were suspended in phosphate buffered saline and adjusted at various pH values (2, 5, 7, 9, and 12) using either 6 M NaOH or 6 M HCl and incubated for 1 h.

### One step growth curve

Latent period and burst size of both SKA49 and SKA64 was determined using modification of method given by Kropinski^78^. Briefly, an overnight culture of QZJM25 was diluted (1:100) in 5 ml LB broth and allowed to grow until its OD600 reached log phase (0.3–0.4) at 37 °C. Later this culture was adjusted to 10^6^ bacterial cells (CFU/ml) in 1 ml prewarmed LB medium and mixed with respective phage at MOI1 (10^6^ pfu) and left without shaking for 30 min to allow phage adsorption. Next the culture was centrifuged (Centrifuge, Helmer, Germany Cat No. ZK 496) at 10,000 rpm for 5 min. Supernatant was removed and stored in a clean tube and titrated to calculate unabsorbed phages, whereas bacterial pellet was resuspended in pre-warmed 30 ml fresh LB in clean flask and allowed to grow. 1 ml aliquots were taken from the culture and immediately centrifuged and supernatant was filtered and saved on ice until titrated. The burst size of phages was calculated using standard formula given in supplementary Table S5.

### Host range of phages

Host range of both phages was determined with a modification of agar overlay spot method illustrated by Adams in 1959^79^. All strains showing zones of lysis were further tested by serial two-fold phage dilutions to determine the ability of each strain to allow growth of phages. All strains isolated during this study were used for host range analysis (Table1). Briefly an overnight culture of each strain was grown in 5 ml LB medium. Next day 250 µl of overnight culture of each strain were mixed with 2.5 ml of soft agar (LB containing 0.5% agar) and poured on to LB agar plates already solidified and warmed at 37 °C. Soft agar was allowed to solidify for 20 min and 10 µl drops of phage lysate were placed on them. After drops absorbed, plates were inverted and incubated at 37 °C overnight. Next day plates were monitored for appearance of zones of lysis as indicator of strain sensitivity to phages.

### Growth reduction assay of *Escherichia coli* strain QZJM25

To test the lysis potential of both phages bacterial growth reduction assay was performed^80^. The overnight culture of QZJM25 was diluted to a final concentration of 1 × 10^3^ CFU/ml in 1 ml and mixed with respective phages SKA49 and SKA64 at MOI 1(10^3^ pfu) and MOI 100 (10^5^ pfu) in triplicates. This mixture was incubated at 37 °C for 30 min to allow efficient phage adsorption. After 30 min this mixture was added to 40 ml LB broth in 100 ml flask for each phage at both MOIs. One flask was left as bacterial control. This experiment was carried out in triplicates. All flasks were incubated at 37 °C for 24 h. Every 2 h, a 1 ml aliquot was removed from each flask and its optical density (600 nm) was measured with spectrophotometer (Cat No. 721-100G, Hinotek, Ningbo, China). A standard curve of QZJM25 CFU/ ml at various optical density values (OD_600_) was used to determine bacterial colony-forming units (CFU) at each growth point (supplementary Figure S1). This curve was used to determine the CFU/ml of QZJM25 in growth reduction assay. Average CFU/ml obtained from three replicates of assay were plotted in graph and statistical significance of results was determined (Fig. 2).

### Genome sequencing and phylogenetic comparisons of phages

Whole genomes of phages were sequenced using Illumina MiSeq platform at Massey Genome Service (Massey University, Palmerston North, New Zealand). NexteraTM XT library kit_V2 (Illumina, San Diego, CA, USA) was used to prepare phage DNA library. Random fragments of phage DNA were produced by enzymatic digestion. Sequencing resulted in 301,237 pair-end (PE) reads with an average read length of 151 bps using software default settings unless otherwise stated. FastQC^81^ (version 0.11.3) was used to perform quality control before and after trimming with Trimmomatic (Version 0.39)^82^. Software PlasmidSPADES (version 3.13.2)^83^was used to assemble genomes and assembly graphs were inspected using Bandage (v 0.8.1) software. Quality of genome assembly was determined using QUAST (version 5.0)^84^ and SQUAT (Dec 2019). GAMOLA2^85^, and RAST software^86^ (server is available at http:// RAST. nmpdr. org) were used to automatically annotate genome. The genomes obtained are uploaded to NCBI GenBank with accession codes OL741059 and OM362897.1.

To calculate phage intergenomic similarities with other members in BLASTn, VIRIDIC software^87^ was used. This software is recommended by ICTV (International Committee on Taxonomy of Viruses), subcommittee of Bacterial and Archaeal viruses (BAVS) and it applies BAVS traditional algorithms to calculate phage genome similarities. VIRIDIC gives best agreement with traditional algorithm that uses percentage identity of two genomes calculated by BLASTN (http://rhea.icbm.uni-oldenburg.de/VIRIDIC/). Easyfig software (freely available at https://mjsull.github.io/Easyfig/) was used to generate genome comparison figure of phages.

To determine suitability of both phages as probable candidates for bacterial control their genomes were tested for presence of lysogenic/temperate lifecycle or recombinase genes. They were also tested for presence of virulence/toxins/antimicrobial resistance markers using PhageLeads software available free at www.phageleads.dk^88^.

The phylogenetic analysis was carried out by the VICTOR web service (https://victor.dsmz.de), a method for the genome-based phylogeny and classification of prokaryotic viruses^89^. All pairwise comparisons of the nucleotide sequences were conducted using the Genome-BLAST Distance Phylogeny (GBDP) method^90^ under settings recommended for prokaryotic viruses. The resulting intergenomic distances were used to infer a balanced minimum evolution tree via FASTME including SPR postprocessing^91^ using the optimal formulas D0. Tree was rooted at the midpoint^92^ and visualized with ggtree^93^. Taxon boundaries at the species, genus and family level were estimated with the OPTSIL program (https://cran.r-project.org/web/packages/optpart/optpart.pdf), using the recommended clustering thresholds and an F value (fraction of links required for cluster fusion) of 0.5^90^. In addition to this a phylogenetic tree based on proteome analysis of both phages was constructed using ViPTree software^94^ freely available at https://www.genome.jp/viptree/. To strengthen the phylogenetic relationship of both SKA49 and SKA64 and verify its classification CoreGenes 5.0 software was used to identify common genes shared with their close homologs^95^. The software is freely available at https://coregenes.ngrok.io/. The amino acid sequence of two orthologs, Major capsid protein (SKA49) and Tail fiber protein (SKA64) identified by CoreGenes analysis were used to construct maximum likelihood tree (MLT). Briefly Major Capsid protein (MCP) and Tail Fiber protein (TFP) amino acid sequences were aligned together in Geneious (v.8.1.9)^96^. The alignments in Phylip format were imported in IQtree^97^ online version^98^ available at http://iqtree.cibiv.univie.ac.at/ to reconstruct a Maximum Likelihood tree based on the best fit model as implemented in the IQTree, using 100 bootstrap value. IQTree implements Model Finder^99^ to calculate best fit model on the data. The trees in Newick format were refined in the online TreeDyn tool^100^ available at http://www.phylogeny.fr/one_task.cgi? task_type = TreeDyn and downloaded in pdf format.

### Statistical analysis

The data analyses were carried out using XLSTAT free 2021 (Addinsoft, Paris–France) and OriginPro 2022 (OriginLab Corporation, Northampton, MA, USA). Depending upon the type of study, the significant difference between variables was assessed using either a student’s t-test or an ANOVA with a *P* value of 0.05 or less (*p* ≤ 0.05) was considered statically significant.

### Ethics approval

*E coli* isolates from urine samples included in this study for comparisons were collected in a previous project approved by institutional ethical review board with letter number CUI/Bio/ERB/6–18/02. All samples were collected after informed patient consent on a pre-approved proforma in native language. Samples were collected following Helsinki guidelines https://www.wma.net/policies-post/wma-declaration-of-helsinki-ethical-principles-for-medical-research-involving-human-subjects/ .

## Supplementary Information

Supplementary Information.

Supplementary Information.

## Data Availability

Accession codes: All sequences generated and presented in this manuscript are uploaded in NCBI genome database under (GenBank) Accession codes; OL741059 can be accessed at https://www.ncbi.nlm.nih.gov/nuccore/OL741059.1/ , OM362897.1 can be accessed at https://www.ncbi.nlm.nih.gov/nuccore/OM362897.1/ and OK086691 can be accessed at https://www.ncbi.nlm.nih.gov/nuccore/OK086691.1/ .
