# Supplementary material for: Characterization of two novel lytic bacteriophages having lysis potential against MDR avian pathogenic Escherichia coli strains of zoonotic potential
Source: Sci Rep. 2023 Jun 20;13:10043. doi: 10.1038/s41598-023-37176-z (PMC10282059; doi:10.1038/s41598-023-37176-z)
Supplement: Supplementary file 1 — Supplementary Information. [file 41598_2023_37176_MOESM1_ESM.docx]

**Table S1. Phylogroups, Virulence genes and MAR indices of *E. coli* isolates of both human and poultry samples**

| **Ser. no** | **Isolate ID** | **Virulence factors identified in *E. coli* isolates of human origin** | **Phylogroup** |
| --- | --- | --- | --- |
| 1 | *E. coli* H2 | *ecp*A *fim*H *tra*T | Average MAR index **ǂ** (0.73)    A (40%) |
| 2 | *E. coli* H3 | *papC* *ecpA* *fim*H traT |  |
| 3 | *E. coli* H8 | *papC* *ecpA* *fim*H *traT* |  |
| 4 | *E. coli* H9 | *papC* *ecpA* *fim*H *traT* |  |
| 5 | *E. coli* H11 | *OmpT* *papC* *ecpA* *fimH traT* |  |
| 6 | *E. coli* H13 | *Vat* *ecpA* *fimH traT* |  |
| 7 | *E. coli* H1 | *OmpT* *papC* *ecpA* *fimH* *traT Aggr* | Average MAR index (0.54)  B2 (33.3 %) |
| 8 | *E. coli* H27 | *Vat* *papC* *ecpA* *fimH* |  |
| 9 | *E. coli* H15 | *Vat* *papC* *ecpA* *fimH traT* |  |
| 10 | *E. coli* H16 | *Vat* *ecpA* *fimH* |  |
| 11 | *E. coli* H22 | *Vat* *papC* *ecpA* *fimH* |  |
| 12 | *E. coli* H5 | *PapC* *ecpA* *fimH traT* | D (26.6 %)  Average MAR index (0.73) |
| 13 | *E. coli* H6 | *ecpA* *fimH traT stx1* |  |
| 14 | *E. coli* H19 | *ecpA* *fimH traT* |  |
| 15 | *E. coli* H12 | *Vat* *papC* *ecpA* *fimH traT* |  |
| **S. no** | **Isolate ID** | **Virulence factors identified in *E coli* isolates of Poultry origin** | **Phylogroup** |
| 1 | *E. coli* P7 | *OmpT* *Aggr* *ecpA* *fimH traT* | D (34.6 %)  Average MAR index (0.782) |
| 2 | *E. coli* P19 | *Aggr* *ecpA* *fimH traT* |  |
| 3 | *E. coli* P23 | *OmpT* *Aggr* *papC* *ecpA* *fimH traT* |  |
| 4 | *E. coli* P27 | *OmpT* *papC* *ecpA* *fimH traT* |  |
| 5 | *E. coli* P28 | *OmpT* *Aggr* *ecpA* *fimH traT* |  |
| 6 | *E. coli* P39 | *PapC* *ecpA* *fimH traT* |  |
| 7 | *E. coli* P49 | *Aggr* *papC ecpA* *fimH traT* |  |
| **8** | ****E. coli* P25**  **(QZJM25)** | ***Aggr* *papC* *ecpA* *fimH traT*** |  |
| 9 | *E. coli* P33 | *OmpT* Aggr *papC* *ecpA* *fimH* *traT* |  |
| 10 | *E. coli* P63 | *Aggr* *papC* *ecpA* *fimH traT* | B1 (23.1%)  Average MAR index (0.83) |
| 11 | *E. coli* P64 | *OmpT* *Aggr ecpA* *fimH traT* |  |
| 12 | *E. coli* P66 | *Aggr* *papC* *ecpA* *fimH traT* |  |
| 13 | *E. coli* P68 | *Aggr* *papC* *ecpA* *fimH traT* |  |
| 14 | *E. coli* P70 | *papC* *fimH traT* |  |
| 15 | *E. coli* P73 | *OmpT* *Vat* *papC* *ecpA* *fimH traT* |  |
| 16 | *E. coli* P6 | *papC* *ecpA* *fimH traT* | A (19.2%)  Average MAR index (0.78) |
| 17 | *E. coli* P34 | *ecpA* *fimH traT* |  |
| 18 | *E. coli* P47 | *Aggr* *papC* *ecpA* *fimH traT* |  |
| 19 | *E. coli* P48 | *papC* *ecpA* *fimH traT* |  |
| 20 | *E. coli* P45 | *OmpT* *papC* *ecpA* *fimH traT* |  |
| 21 | *E. coli* P1 | *Vat* *Aggr* *papC* *ecpA* *fimH traT* | Clade I (15.4%)  Average MAR index (0.86) |
| 22 | *E. coli* P2 | *OmpT* *Vat* *Aggr* *ecpA* *fimH traT* |  |
| 23 | *E. coli* P36 | *ecpA* *fimH traT* |  |
| 24 | *E. coli* P46 | *OmpT* *papC* *ecpA* *fimH traT* |  |
| 25 | *E. coli* P51 | *PapC ecpA* *fimH traT* | F (3.8%) |
| 26 | *E. coli* P69 | *papC* *ecpA* *fimH traT* | B2 (3.8 %) |

*****E. coliP25 isolate is later named as QZJM25 (OK086691) and is used for isolation of bacteriophages resistance profile of QZJM25 is given in supplementary table 2

**ǂ MAR index = No of antibiotics an isolate is resistant**

**Total No of antibiotics tested.**

**Table S2. Resistance profile of *E. coli* isolates of both human and poultry origin**

| **S. #** | **Antibiotics Class** | **Antibiotic** | **CODE** | **Concentration (µg/disc)** | **(%) Resistant**  ***E. coli* H *E. coli* P (n=15) (n=26)** | |
| --- | --- | --- | --- | --- | --- | --- |
| 1 | Aminoglycosides | Neomycin | NEO | 30µg/ml | 82% | 81% |
| 2 |  | Kanamycin | Kn | 30 µg/ml | 41% | 70% |
| 3 |  | Streptomycin | STR | 25µg/ml | 53% | 93% |
| 4 | Sulphonamide/ Folic Acid Inhibitors | Sulphamethoxazole | SXT | 25µg/ml | 65% | 85% |
| 5 |  | Trimethoprim | W | 30µg/ml | 82% | 89% |
| 6 | Beta-Lactams | Ampicillin | AMP | 10µg/ml | 100% | 100% |
| 7 | Cephalosporins | Cefotaxime | CTX | 30µg/ml | 100% | 15% |
| 8 | Quinolone | Nalidixic Acid | NAL | 30µg/ml | 70.5% | 96% |
| 9 | Tetracycline | Tetracycline | TET | 30µg/ml | 71% | 100% |
| 10 | Phenicol | Chloramphenicol | C | 30 µg/ml | 35% | 81% |
| 11 | Macrolide | Erythromycin | ERY | 15µg/ml | 94% | 100% |

**Table S3;** **Resistance profile of QZJM25 *Escherichia coli* host strain used for bacteriophage isolation.**

| Antibiotics Class | Antibiotics | Code | Concentration | Zone diameter (mm) | Sensitivity |
| --- | --- | --- | --- | --- | --- |
|  |  |  | µg/ml |  |  |
| β-Lactams | Penicillin G | P | 10 | ----- | R ǂ |
|  | Amoxycillin | AML | 10 | ----- | R |
|  | Amoxycillin-clavulanic acid | AMC | 30 | ----- | R |
|  | Amoxycillin | AML | 25 | ------ | R |
|  | Imipenem | IPM | 10 | ----- | R |
|  | Meropenem | MEM | 10 | 23mm | I* |
|  | Piperacillin+ tazobactam | TPZ | 110 | 19mm | I |
|  | Ampicillin | AM | 10 | ----- | R |
|  | Flucloxacillin | FL | 5 |  | R |
|  | Cefoperazone/ sulbactam | CES | 105 | 20mm | I |
|  | Ceftazidime | CAZ | 30 | ------ | R |
| Aminoglycosides | Streptomycin | S | 10 | 16mm | S ֎ |
|  | Gentamicin | CN | 10 | 6mm | R |
|  | Kanamycin | K | 30 | 11 mm | R |
| Tetracyclines | Tetracycline | TE | 30 | ----- | R |
| Quinolones | Levofloxacin | LEV | 5 | 9mm | R |
|  | Norfloxacin | NOR | 10 | 9mm | R |
|  | Nalidixic acid | NA | 30 | ------ | R |
|  | Ciprofloxacin | CIP | 5 | 8mm | R |
|  | (Fluoroquinolone) |  |  |  |  |
| Glycopeptides | Vancomycin | VA | 30 | 8mm | R |
| Phenicols | Chloramphenicol | C | 30 | 24mm | S |
| Macrolides | Azithromycin | AZM | 15 | 17mm | S |
|  | Erythromycin | E | 15 | ----- | R |
| Sulphonamides | Sulphamethoxazole/ | SXT | 25 | 22mm | S |
|  | Trimethoprim |  |  |  |  |
| Lincosamides | Lincomycin | L | 2 | ----- | R |
| Aminocoumarin | Novobiocin | NV | 30 | ----- | R |
| Ansamycin | Rifampicin | RD | 5 | 11 | R |

**ǂ R= resistant *I= intermediate resistance ֎ S= sensitive**

**Table S4. Primers used in this study for pathotyping and phylotyping of *E. coli* isolates.**

| **Gene** | **Primer**  **Nucleotide sequence (5´-3´)** | **Size (bp)** | **Reference** |
| --- | --- | --- | --- |
| *aggR* | GTATACACAAAAGAAGGAAGC  ACAGAATCGTCAGCATCAGC | 254 | (1) |
| *eae* | TCAATGCAGTTCCGTTATCAGTT  GTAAAGTCCGTTACCCCAACCTG | 482 |  |
| *ST* | AATTGCTACTATTCATGCTTTCAGGAC  TCTTTTTCACCTTTC GCTCAGG | 133 | (2) |
| *Stx1* | ACA CTG GAT GAT CTC AGT GG  CTG AAT CCC CCT CCA TTA TG | 614 | (3) |
| *Stx1* | GAATTTACCTTAGACTTCTCGAC  TCCTGTTAACAAATCCTGTCAC | 250 |  |
| *Stx2* | CCATGA CAA CGG ACA GCA GTT  CCT GTC AAC TGA GCA CTT TG | 779 |  |
| *cnf1* | GCTCAACGAGACTATGCTCTG  ACGCTGCTAAGTACCTCCTGG | 278 | (4) |
| *vat* | GTATATGGGGGGCAACATAC  GTGTCAGAACGGAATTGTCG | 1337 | (5) |
| *ompT* | ATCTAGCCGAAGAAGGAGGC  CCCGGGTCATAGTGTTCATC | 559 | (6) |
| *iroN* | AAGTCAAAGCAGGGGTTGCCCG  GACGCCGACATTAAGACGCAG | 667 |  |
| *ecpA* | TGA AAA AAA AGG TTC TGG CAA TAG C  CGCTGA TGA GGA GAA AGT GAA | 541 | (7) |
| *papC* | GTGGCAGTATGA GTA ATG ACC GTT A  ATATCC TTT CTG CAG GGA TGC AAT A | 200 |  |
| *fimH* | TGC AGA ACG GAT AAG CCG TGG  GCA GTC ACC TGC CCT CCG GTA | 508 |  |
| *traT* | GGT GTG GTG CGA TGA GCA CAG  CAC GGT TCA GCG ATC CCT GAG | 290 |  |
| *sfa/*  *fogDE* | CTC CGG AGA ACT GGG TGC ATC TTA C  CGG AGG AGT AAT TAC AAA CCT GGC A | 410 |  |

| **Gene** | **Nucleotide sequence (5´-3´)** | **Size (bp)** | **Reference** |
| --- | --- | --- | --- |
| *chuA* | ATGGTACCGGACGAACCAAC  TGCCGCCAGTACCAAAGACA | 288 | (8) |
| *yjaA* | CAAACGTGAAGTGTCAGGAG  AATGCGTTCCTCAACCTGTG | 211 |  |
| *TspE4.C2* | CACTATTCGTAAGGTCATCC  AGTTTATCGCTGCGGGTCGC | 152 |  |
| *arpA* | AACGCTATTCGCCAGCTTGC  TCTCCCCATACCGTACGCTA | 400 |  |

**Table S5; Calculations of Burst size for *Escherichia* phage SKA49 and *Escherichia* phage SKA64**

| **Phage ID** | **Replicate** | **Total applied phages (A)ǂ** | **Free phages (B)** | **Phages after burst (C)** | **Burst size (D/E) *** |
| --- | --- | --- | --- | --- | --- |
| ***Escherichia* phage SKA49** | **1** | **1× 10^6^** | **3.1x10^4^** | **5 x10^7^** | **51** |
|  | **2** | **1× 10^6^** | **4.5 x10^5^** | **2.7x10^7^** | **47.3** |
|  | **3** | **1× 10^6^** | **3.6x10^5^** | **4.6x10^7^** | **70** |
| **Average Burst Size** |  |  |  |  | **~ 56 virions /cell** |
| ***Escherichia* phage SKA64** | **1** | **1× 10^6^** | **9.0×10^4^** | **2.1×10^7^** | **22** |
|  | **2** | **1× 10^6^** | **8.6×10^4^** | **3.2×10^7^** | **34** |
|  | **3** | **1× 10^6^** | **8.23×10^4^** | **4.1×10^7^** | **44** |
| **Average Burst Size** |  |  |  |  | **33 virions/cell** |

*Formula: **Burst size** = Phages after burst (C)- Free phages (B) = New phages released (D)

Total applied phages (A)- Free phages (B) Number of infecting phages (E)


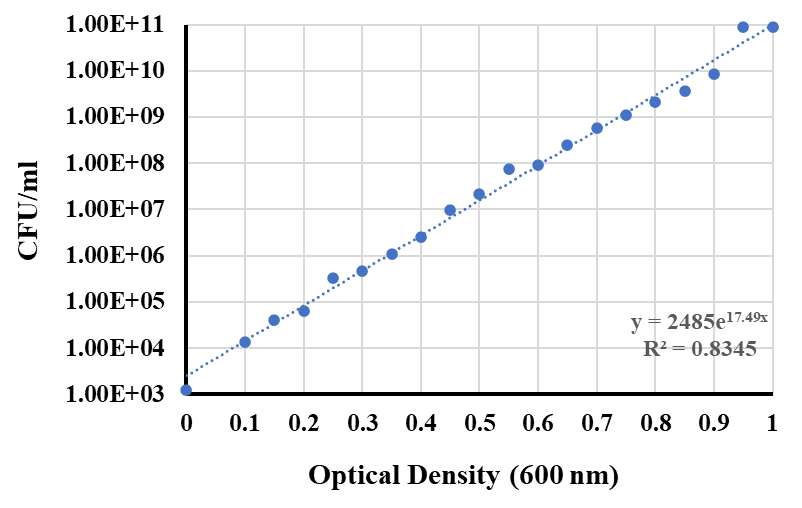


**Figure S1;** Standard curve for QZJM25 growth as a factor of optical density

**Table S6A; Escherichia phage SKA49 Cluster table generated by VIRIDIC**

| Phage genomes | Species cluster | genus cluster |
| --- | --- | --- |
| Yersinia phage PYps16T | 7 | 1 |
| Yersinia phage PYps23T | 5 | 1 |
| Yersinia phage PYps16N | 6 | 1 |
| **Escherichia-Phage-SKA49** | **2** | **1** |
| Escherichia phage Mangalitsa | 9 | 1 |
| Escherichia phage phiEcoM-GJ1 | 1 | 1 |
| Escherichia phage flopper | 10 | 1 |
| Escherichia phage vB_EcoP_Bp7 | 3 | 1 |
| Escherichia phage vB_EcoM_Bp10 | 4 | 1 |
| Escherichia phage ST32 | 8 | 1 |
| Escherichia phage vB_EcoM_SA91KD | 11 | 1 |

| Phage Genomes | species_ cluster | genus_ cluster |
| --- | --- | --- |
| Escherichia phage nieznany | 9 | 1 |
| Escherichia phage tuntematon | 10 | 1 |
| Escherichia phage nepoznato | 8 | 1 |
| Escherichia phage ESCO5 | 4 | 1 |
| Escherichia phage vB_EcoM-Ro121c4YLVW | 6 | 1 |
| Escherichia phage vB_EcoM_Schickermooser | 2 | 1 |
| Escherichia phage ESCO13 | 2 | 1 |
| Escherichia phage phAPEC8_ev052 | 2 | 1 |
| Escherichia phage vB_EcoM-Ro121lw | 2 | 1 |
| **Escherichia phage SKA64** | **13** | **1** |
| Escherichia phage phAPEC8 | 1 | 1 |
| Escherichia phage PNJ1809-36 | 1 | 1 |
| Escherichia phage ukendt | 11 | 1 |
| Klebsiella phage ZCKP1 | 5 | 1 |
| Escherichia phage vB_EcoM_UP17 | 3 | 1 |
| Dompiswa phage TSP7_1 | 3 | 1 |
| Escherichia phage anhysbys | 7 | 1 |
| phage Mt1B1_P17 | 12 | 1 |
| Escherichia phage BI-EHEC | 12 | 1 |

**Table S6B; Escherichia phage SKA64 Cluster table generated by VIRIDIC**

**Table S7; SKA49 predicted ORFs and their amino acid homology with closest relatives (BLAST P)**

| **ORF** | **nucleotide seq** | **Total AA** | **Putative Function** | **Query coverage** | **Similarity** | **GB Ac. NO** | **Organism** |
| --- | --- | --- | --- | --- | --- | --- | --- |
|  | 2115bp | 704AA | Putative RNA polymerase | 98% | 98.15% | QEM42537.1 | *Escherichia* phage vB_EcoM_Bp10 |
|  | 129bp | 42AA | Hypothetical protein | 97% | 95.24% | QQO91003.1 | Yersinia phage PYps3T |
|  | 174bp | 57AA | Hypothetical protein | - | - | - | - |
|  | 249bp | 82AA | hypothetical protein | 98% | 91.46% | YP_009850474.1 | *Escherichia* phage Mangalitsa |
|  | 243bp | 80AA | hypothetical Protein (putative EaE protein) | 98% | 91.25% | QEM42569.1 | *Escherichia* phage vB_EcoM_Bp10 |
|  | 189bp | 62AA | hypothetical protein | 98% | 98.39% | YP_009790665.1 | *Escherichia* phage ST32 |
|  | 165bp | 54AA | hypothetical protein | 98% | 98.15% | QQO90928.1 | Yersinia phage PYps23T |
|  | 183bp | 60AA | Hypothetical protein | 88% | 40.74% | YP_009790666.1 | *Escherichia* phage ST32 |
|  | 174bp | 57AA | Hypothetical protein | 98 | 94.74% | YP_009790668.1 | *Escherichia* phage ST32 |
|  | 297bp | 98AA | hypothetical protein | 98% | 70.41% | YP_009850479.1 | *Escherichia* phage Mangalitsa |
|  | 264bp | 87AA | hypothetical protein | 98% | 100.00% | YP_009855568.1 | *Escherichia* phage flopper |
|  | 228bp | 75AA | Hypothetical protein | 98% | 97.33% | YP_009790672.1 | *Escherichia* phage ST32 |
|  | 291bp | 96AA | hypothetical protein | 98% | 100.00% | YP_009790674.1 | *Escherichia* phage ST32 |
|  | 234bp | 77AA | Hypothetical protein | 98% | 85.71% | YP_009855564.1 | *Escherichia* phage flopper |
|  | 288bp | 95AA | hypothetical protein | 98% | 98.95% | YP_009790676.1 | *Escherichia* phage ST32 |
|  | 153bp | 50AA | Hypothetical protein | 98% | 92% | QQO91017.1 | Yersinia phage PYps3T |
|  | 381bp | 126AA | single-stranded DNA-binding protein | 99% | 90.48% | YP_001595410.1 | *Escherichia* phage phiEcoM-GJ1 |
|  | 477bp | 158AA | hypothetical protein | 99% | 91.77% | YP_009850487.1 | *Escherichia* phage Mangalitsa |
|  | 351bp | 116AA | hypothetical protein | 99% | 99.14% | YP_009790681.1 | *Escherichia* phage ST32 |
|  | 234bp | 77AA | Hypothetical Protein | 99% | 100% | UIU26832.1 | *Escherichia* phage vB_EcoM_SA91KD |
|  | 267 | 88AA | Hypothetical protein | 98% | 90.91% | YP_009850491.1 | *Escherichia* phage Mangalitsa |
|  | 417bp | 138AA | Imidazole glycerol phosphate synthase, Putative endolysin | 99% | 99.28% | YP_009790684.1 | *Escherichia* phage ST32 |
|  | 546bp | 181AA | putative anti-restriction protein | 99% | 82.87% | YP_001595417.1 | *Escherichia* phage phiEcoM-GJ1 |
|  | 240bp | 79AA | Hypothetical protein | 98% | 98.73% | YP_009790687.1 | *Escherichia* phage ST32 |
|  | 318bp | 105AA | hypothetical protein | 99% | 74.29 | YP_009850499.1 | Yersinia phage PYps16N |
|  | 309bp | 102AA | Hypothetical protein | 99% | 100% | YP_009850500.1 | *Escherichia* phage Mangalitsa |
|  | 675bp | 224AA | hypothetical protein | 99% | 100.00% | YP_001595423.1 | *Escherichia* phage phiEcoM-GJ1 |
|  | 339bp | 112AA | hypothetical protein | 99% | 99.11% | YP_001595424.1 | *Escherichia* phage phiEcoM-GJ1 |
|  | 192bp | 63AA | Putative endolysin | 84% | 81.48% | QQO91037.1 | Yersinia phage PYps3T |
|  | 141bp | 46AA | conserved hypothetical_179 | 97% | 100.00% | YP_009790695.1 | *Escherichia* phage ST32 |
|  | 237bp | 78AA | Phage protein putative endolysin, a spannin | 98% | 93.59% | YP_009850507.1 | *Escherichia* phage Mangalitsa |
|  | 231bp | 76AA | Putative tail tape measure protein | 98% | 98.68% | YP_009790697.1 | *Escherichia* phage ST32 |
|  | 651bp | 216AA | thymidylate synthase, flavin-dependent, thyX_182 | 99% | 99.54% | QDJ96616.1 | *Escherichia* phage vB_EcoP_Bp7 |
|  | 1764bp | 587AA | replicative DNA helicase, dnaB_183 | 99% | 100.00% | YP_009790699.1 | *Escherichia* phage ST32 |
|  | 1935bp | 644AA | DNA polymerase | 99% | 98.45% | YP_001595432.1 | *Escherichia* phage phiEcoM-GJ1 |
|  | 264bp | 81AA | Hypothetical protein | 98% | 98.73% | YP_009790687.1 | *Escherichia* phage ST32 |
|  | 867bp | 288AA | hypothetical protein | 99% | 99.65% | YP_001595434.1 | *Escherichia* phage phiEcoM-GJ1 |
|  | 429bp | 142AA | Base plate wedge protein | 99% | 95.07% | QQO90963.1 | Yersinia phage PYps23T |
|  | 1026bp | 341AA | 5’-3’ exonuclease, RNaseH  DNA polymerase I, polA_188 | 99% | 92.67% | YP_009850517.1 | *Escherichia* phage Mangalitsa |
|  | 525bp | 174AA | Putative Replicative endonuclease VII | 99% | 99.43% | QDJ96622.1 | *Escherichia* phage vB_EcoP_Bp7 |
|  | 756bp | 251AA | DNA ligase D, ligase domain, ligD_190 | 99% | 99.20% | YP_001595437.1 | *Escherichia* phage phiEcoM-GJ1 |
|  | 627bp | 208AA | Putative adénylate kinase | 99% | 99.52% | YP_009855608.1 | *Escherichia* phage flopper |
|  | 600bp | 199AA | deoxyuridine 5'-triphosphate nucleotide hydrolase (dUTP ) | 99% | 84.21% | YP_009790707.1 | *Escherichia* phage ST32 |
|  | 330bp | 109AA | Hypothetical protein | 99% | 99.25% | YP_001595440.1 | *Escherichia* phage phiEcoM-GJ1 |
|  | 186bp | 61AA | Hypothetical protein | 98% | 98.36% | YP_009850523.1 | *Escherichia* phage Mangalitsa |
|  | 2019bp | 672AA | Putative terminase large subunit | 99% | 100.00% | QDJ96627.1 | *Escherichia* phage vB_EcoP_Bp7 |
|  | 213bp | 70AA | Major head subunit precursor protein | 98% | 97.14% | QQO91059.1 | Yersinia phage PYps3T |
|  | 1317bp | 438AA | Portal protein | 99% | 100.00% | YP_009790713.1 | *Escherichia* phage ST32 |
|  | 1065bp | 355AA | Major head subunit precursor protein | 99% | 98.5% | YP_001595446.1 | *Escherichia* phage phiEcoM-GJ1 |
|  | 474bp | 157AA | hypothetical protein | 99% | 98.09% | YP_009790715.1 | *Escherichia* phage ST32 |
|  | 213bp | 70AA | Hypothetical protein | 98% | 88.57 | QDJ96593.1 | *Escherichia* phage vB_EcoP_Bp7 |
|  | 1008bp | 335AA | major capsid protein | 99% | 98.21% | QEM42515.1 | *Escherichia* phage vB_EcoM_Bp10 |
|  | 441bp | 146AA | Putative head tail adaptor protein | 99% | 100.00% | QEM42516.1 | *Escherichia* phage vB_EcoM_Bp10 |
|  | 390bp | 129AA | Tail complétion protein | 99% | 100.00% | QDJ96634.1 | *Escherichia* phage vB_EcoP_Bp7 |
|  | 363bp | 120AA | Putative Head tail adapter protein | 99% | 100% | YP_001595451.1 | *Escherichia* phage phiEcoM-GJ1 |
|  | 513bp | 170AA | Putative tail completion protein | 99% | 98.24% | YP_009790721.1 | *Escherichia* phage ST32 |
|  | 1449bp | 482AA | Tail sheath protein | 99% | 97.10% | YP_009850535.1 | *Escherichia* phage Mangalitsa |
|  | 456bp | 151AA | Tail tube protein | 99% | 99.3% | YP_009850536.1 | *Escherichia* phage Mangalitsa |
|  | 459bp | 152AA | Putative tail assembly chaperon protein | 99% | 99.34% | YP_001595455.1 | *Escherichia* phage phiEcoM-GJ1 |
|  | 3276bp | 1241AA | Putative tail fiber protein | 99% | 99.11% | YP_009790726.1 | *Escherichia* phage ST32 |
|  | 1095bp | 364AA | Putative Minor baseplate protein | 99% | 98.35% | YP_009850539.1 | *Escherichia* phage Mangalitsa |
|  | 903bp | 300AA | Tail tube initiator protein | 99% | 98.67% | YP_009850542.1 | *Escherichia* phage Mangalitsa |
|  | 369bp | 123AA | Minor baseplate protein | 99% | 96.72% | YP_009850541.1 | *Escherichia* phage Mangalitsa |
|  | 795bp | 264AA | Putative base plate assembly protein | 99% | 97.73% | YP_009850542.1 | *Escherichia* phage Mangalitsa |
|  | 372bp | 123AA | Tail tube polymerization initiator protein | 98% | 97.54% | YP_009850543.1 | *Escherichia* phage Mangalitsa |
|  | 1158bp | 385AA | Base plate wedge protein | 99% | 96.88% | YP_009850544.1 | *Escherichia* phage Mangalitsa |
|  | 642bp | 213AA | Base plate wedge protein | 99% | 92.5% | YP_009850545.1 | *Escherichia* phage Mangalitsa |
|  | 1146bp | 381AA | Putative tail fiber protein | 99% | 93.96% | YP_009850546.1 | *Escherichia* phage Mangalitsa |
|  | 1392bp | 463AA | Phage minor tail protein | 99% | 91.59% | YP_009850547.1 | *Escherichia* phage Mangalitsa |
|  | 1074bp | 357AA | putative tail fiber protein | 99% | 69.25% | YP_009850548.1 | *Escherichia* phage Mangalitsa |
|  | 342bp | 113AA | phage holin, lambda family | 99% | 99.12% | YP_009790737.1 | *Escherichia* phage ST32 |
|  | 555bp | 184AA | Spore cortex lytic enzyme (Putative cell wall hydrolase) | 100% | 99.32% | YP_009855578.1 | *Escherichia* phage flopper |
|  | 1101 | 366AA | ribonucleoside-diphosphate reductase, class 1b, | 99% | 99.18% | YP_009855577.1 | *Escherichia* phage flopper |

**
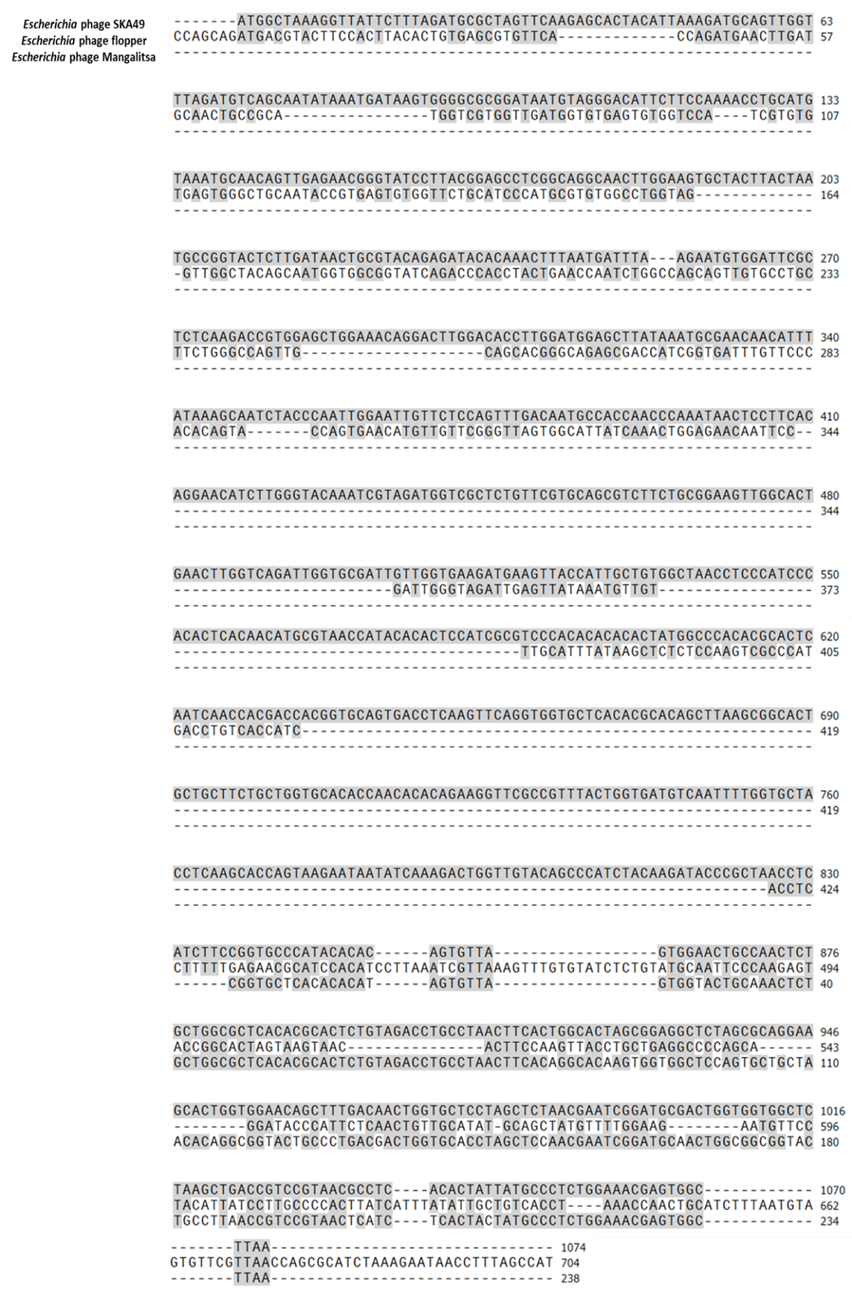
**

**Figure S2; Nucleotide sequence comparison of tail fiber genes of SKA49 homologs. Name of pages given on top left hand. Gray shading indicates nucleotides that resemble SKA49**

**Table S8; SKA64 predicted ORFs and their amino acid homology with closest relatives (BLAST P)**

| **ORF** | **ORF (bp)** | **Total AA** | **Putative Function** | **Query coverage** | **Similarity** | **GB Ac. NO** | **Organism** |
| --- | --- | --- | --- | --- | --- | --- | --- |
| 1 | 2076 | 691 | phage terminase large subunit | 99% | 100% | YP_009985582.1 | Escherichia phage anhysbys |
| 2 | 393 | 130 | phage lysis regulatory protein, LysB family_34 | 99% | 100% | YP_007348513.1 | Escherichia phage phAPEC8 |
| 4 | 297 | 98 | RNA polymerase sigma factor |  |  |  |  |
| 11 | 354 | 117 | putative transposase-like protein | 99% | 100% | CAA7332674.1 | Escherichia phage vB_EcoM_UP17 |
| 16 | 609 | 202 | putative DNA binding protein | 99% | 87.62% | DAU99217.1 | Myoviridae sp. |
| 20 | 552 | 183 | Metallo-phosphoesterase, DNA ligase-associated protein | 99% | 100% | QPI12963.1 | Escherichia phage PNJ1809-36 |
| 21 | 933 | 310 | putative RNA ligase/RNA repair | 99% | 100% | YP_010356424.1 | Dompiswa phage TSP7_1 |
| 22 | 405 | 134 | dephospho-CoA kinase, coaE_54 | 99% | 100% | YP_009803359.1 | Klebsiella phage ZCKP1 |
| 26 | 1116 | 371 | DNA ligase I, ATP-dependent (dnl1) | 99% | 100% | YP_009987099.1 | Escherichia phage Mt1B1_P17 |
| 29 | 726 | 241 | Putative NTP phosphohydrolase | 99% | 100% | WP_016069135.1 | Escherichia coli |
| 30 | 879 | 292 | putative exonuclease RdgC | 99% | 100% | WP_229510944.1 | Escherichia coli |
| 32 | 690 | 229 | putative exonuclease | 99% | 99.56% | YP_009986143.1 | Escherichia phage nieznany |
| 33 | 960 | 319 | DNA polymerase III, epsilon subunit, dnaQ_65 | 99% | 100% | YP_009786566.1 | Escherichia phage ESCO13 |
| 39 | 999 | 332 | thymidylate synthase, flavin-dependent, thyX_71 | 99% | 100% | YP_007348481.1 | Escherichia phage phAPEC8 |
| 42 | 2241 | 746 | ribonucleoside-diphosphate reductase, alpha subunit | 99% | 100% | YP_009986579.1 | Escherichia phage tuntematon |
| 43 | 1086 | 361 | ribonucleoside-diphosphate reductase, class 1b, | 99% | 100% | YP_007348476.1 | [Escherichia phage phAPEC8] |
| 45 | 153 | 50 | chaperone protein DnaJ, dnaJ_77 | 98 | 100 | YP_009986826.1 | Escherichia phage ukendt |
| 48 | 993 | 330 | dTDP-glucose 4,6-dehydratase, rfbB_80 | 99% | 100% | YP_009984956.1 | Escherichia phage vB_EcoM-Ro121c4YLVW |
| 49 | 879 | 292 | glucose-1-phosphate thymidylyltransferase, rfbA_81 | 99% | 100% | YP_009984957.1 | Escherichia phage vB_EcoM-Ro121c4YLVW |
| 50 | 567 | 188 | dTDP-4-dehydrorhamnose 3,5-epimerase, rfbC_82 | 99 | 99.47 | YP_007348469.1 | Escherichia phage phAPEC8 |
| 51 | 843 | 280 | dTDP-4-dehydrorhamnose reductase, rfbD_83 | 99% | 100% | QPI12932.1 | Escherichia phage PNJ1809-36 |
| 52 | 261 | 86 | glutaredoxin 3, grxC_84 | 98% | 98.84% | YP_009787073.1 | Escherichia phage ESCO5 |
| 54 | 501 | 166 | tyrosine 2,3-aminomutase (Lysozyme) | 99% | 100% | YP_009786544.1 | Escherichia phage ESCO13 |
| 55 | 759 | 252 | CRISPR-associated helicase Cas3_87 | 99% | 100% | YP_009786543.1 | Escherichia phage ESCO13 |
| 57 | 693 | 230 | ATP-dependent protease HslVU, peptidase subunit | 99% | 100% | YP_007348462.1 | Escherichia phage phAPEC8 |
| 65 | 744 | 247 | polynucleotide kinase-phosphatase_97 | 99% | 100% | YP_007348454.1 | Escherichia phage phAPEC8 |
| 79 | 801 | 266 | putative Sir2-like protein [Escherichia phage p | 99% | 99.25% | QPI12904.1 | Escherichia phage PNJ1809-36 |
| 88 | 450 | 149 | putative zinc finger/helix-turn-helix protein, | 99% | 100% | WP_016069063.1 | Escherichia coli |
| 89 | 198 | 65 | DNA ligase | 99% | 100% | YP_010356356.1 | Dompiswa phage TSP7_1 |
| 101 | 672 | 223 | Resolvase/ invertase | 99% | 100% | WP_016069063.1 | Escherichia coli |
| 110 | 198 | 65 | Flagellar assembly protein FliH | 98% | 100% | WP_229504070.1 | Escherichia coli |
| 126 | 462 | 153 | transcription-repair coupling factor, mfd_158 | 98% | 99.34% | YP_010356318.1 | Dompiswa phage TSP7_1 |
| 168 | 981 | 326 | Putative H endonuclease | 99% | 75.31% | YP_007348621.1 | Escherichia phage phAPEC8 |
| 169 | 1140 | 379 | DnaA regulatory inactivator Hda, hda_202 | 99% | 99.74% | QPI12812.1 | Escherichia phage PNJ1809-36 |
| 196 | 1593 | 530 | DNA helicase II, uvrD_229 | 99% | 100% | YP_009803461.1 | Klebsiella phage ZCKP1 |
| 198 | 558 | 185 | putative seryl-tRNA synthetase | 99% | 99.46% | YP_007348592.1 | Escherichia phage phAPEC8 |
| 200 | 1815 | 604 | anaerobic ribonucleoside-triphosphate reductase large subunit | 99% | 100% | YP_009985387.1 | Escherichia phage anhysbys |
| 201 | 471 | 156 | anaerobic ribonucleoside-triphosphate reductase small subunit | 99% | 100% | YP_007348589.1 | Escherichia phage phAPEC8 |
| 204 | 657 | 218 | HAD hydrolase, REG-2-like, family IA_237 | 99% | 100% | YP_007348586.1 | Escherichia phage phAPEC8 |
| 205 | 828 | 275 | phosphoribosyl transferase family protein | 99% | 100% | QPI12774.1 | Escherichia phage PNJ1809-36 |
| 213 | 591 | 196 | SprT family zinc-dependent metalloprotease | 99% | 100% | YP_009803445.1 | Klebsiella phage ZCKP1 |
| 224 | 522 | 173 | Spore cortex lytic enzyme SleB family protein | 99% | 100% | YP_007348567.1 | Escherichia phage phAPEC8 |
| 233 | 1779 | 592 | replicative DNA helicase, dnaB_266 | 99% | 100% | YP_007348558.1 | Escherichia phage phAPEC8 |
| 235 | 795 | 264 | putative DNA N6-adenine methyltransferase | 99% | 100% | WP_229504230.1 | Escherichia coli |
| 236 | 2598 | 865 | DNA polymerase I, polA_269 | 99% | 100% | YP_007348555.1 | Escherichia phage phAPEC8 |
| 240 | 594 | 197 | putative tRNA nucleotidyl transferase / poly(A) | 99% | 100% | YP_007348551.1 | Escherichia phage phAPEC8 |
| 243 | 2676bp | 891AA | Tail fiber protein | 99% | 99.78% | YP_009786631.1 | Escherichia phage ESCO13 |
| 244 | 1650 | 549 | putative phage tail fiber protein | 99% | 99.27% | QPI13012.1 | Escherichia phage PNJ1809-36 |
| 245 | 3054 | 1017 | colanic acid biosynthesis protein WcaM, wcaM_1 | 99% | 100% | WP_016069188.1 | Escherichia coli |
| 247 | 546 | 181 | putative tail fiber assembly protein | 99% | 99.45% | YP_009987047.1 | Escherichia phage Mt1B1_P17 |
| 248 | 1062 | 353 | putative gpH domain protein Tail fiber protein | 99% | 99.43% | YP_010356201.1 | Dompiswa phage TSP7_1 |
| 249 | 630bp | 210 | Base plate protein | 99% | 99.52% | YP_009787153.1 | Escherichia phage ESCO5 |
| 250 | 1488 | 495 | Putative base plate wedge protein | 99% | 99.80% | YP_009987050.1 | Escherichia phage Mt1B1_P17 |
| 252 | 345 | 114 | tail spike protein | 73% | 79.76% | YP_009803406.1 | Klebsiella phage ZCKP1 |
| **253** | **2550** | **849** | **Putative tail spike protein** | **91%** | **79.77%** | **QEG09597.1** | **Escherichia phage Pisces** |
| 254 | 2154 | 717 | Non-contractile tail sheath | 99% | 99.58% | WAX14384.1 | non-contractile tail sheath |
| 255 | 2877 | 958 | Tail fiber protein | 99% | 99.90% | YP_009986348.1 | Escherichia phage nieznany |
| 257 | 627 | 208 | base plate wedge protein | 99% | 99.52% | YP_009985601.1 | Escherichia phage anhysbys |
| 258 | 735 | 244 | base plate central spike protein | 99% | 99.59% | YP_009985600.1 | Escherichia phage anhysbys |
| 259 | 1011 | 336 | Putative base plate hub | 99% | 100% | YP_009787144.1 | Escherichia phage ESCO5 |
| 261 | 702 | 233 | Putative tail tube initiator protein | 99% | 99.57% | YP_007348531.1 | Escherichia phage phAPEC8 |
| 262 | 1989 | 662 | putative tail tape measure protein | 99% | 100% | YP_009987060.1 | Escherichia phage Mt1B1_P17 |
| 263 | 483 | 160 | Tail assembly chaperon | 99% | 100% | WP_016069170.1 | Escherichia coli |
| 264 | 480 | 159 | tail tube protein | 99% | 99.37% | YP_009987063.1 | Escherichia phage Mt1B1_P17 |
| 265 | 1374 | 457 | tail sheath protein | 99% | 99.78% | YP_009787138.1 | Escherichia phage ESCO5 |
| 266 | 660 | 219 | Putative tail completion protein | 99% | 100% | YP_009984901.1 | Escherichia phage vB_EcoM-Ro121c4YLVW |
| 267 | 417 | 138 | minor capsid protein | 99% | 100% | YP_007348524.1 | Escherichia phage phAPEC8 |
| 268 | 489 | 162 | virion morphogenesis protein | 99% | 100% | YP_009803387.1 | Klebsiella phage ZCKP1 |
| 269 | 549 | 182 | tail capping protein | 99% | 99.38% | YP_009787135.1 | Escherichia phage ESCO5 |
| 270 | 1002 | 333 | putative major head protein | 99% | 100% | OL739525.1 | Escherichia phage dw-ec |
| 271 | 399 | 132 | putative head stabilization/decoration protein | 99% | 99.24% | YP_009985586.1 | Escherichia phage anhysbys |
| 272 | 1116 | 371 | scscaffold protein | 99% | 99.24% | YP_009985586.1 | Escherichia phage anhysbys |
| 273 | 480 | 159 | serine protease | 99% | 99.37% | YP_009985584.1 | Escherichia phage anhysbys |
| 274 | 1566 | 521 | putative portal protein | 99% | 99.81% | YP_009786600.1 | Escherichia phage ESCO13 |

ORF number corresponds to labelling in genome map given in main manuscript (Figure 7). Only those ORFs are listed that resemble characterized proteins in NCBI.

**Table S9A; Comparison of SKA49 with close homologs of the genus *Carltongylesvirus***

| **Phage** | **Total**  **ORF** | **Genome**  **Size (bp)** | **G+C**  **Content**  **(%)** | **Accession**  **No** | **Identity**  **(%) *** |
| --- | --- | --- | --- | --- | --- |
| ***Escherichia* phage SKA49** | 73 | 51548 | 44% | OL741059.1 | 100% |
| ***Escherichia* phage vB_EcoM_Bp10** | 72 | 52288 | 44% | MN122072.1 | 85.7% |
| ***Escherichia* phage Mangalitsa** | 81 | 52329 | 44% | NC_048799.1 | 84.6% |
| ***Escherichia* phage flopper** | 71 | 52092 | 44% | NC_048845.1 | 84.5% |
| ***Escherichia* phage vB_EcoP_Bp7** | 64 | 52615 | 44% | MN117721.1 | 84.1% |
| ***Escherichia* phage ST32** | 79 | 53092 | 44% | NC_047830.1 | 84% |
| ***Escherichia* phage phiEcoM-GJ1** | 75 | 52975 | 44% | EF460875.1 | 81.5% |
| ***Escherichia* phage vB_EcoM_SA91KD** | 78 | 53673 | 44% | OL960574.1 | 83.4% |

**Table S9B; Comparison of SKA64 with close homologs of the genus**  ***Phapecoctavirus***

| **Phage** | **Total**  **ORF** | **Genome**  **Size (bp)** | **G+C**  **Content**  **(%)** | **Accession**  **No** | **Identity**  **(%) *** |
| --- | --- | --- | --- | --- | --- |
| **Escherichia phage SKA64** | 275 | 152.401Kb | 39% | OM362897.1 | 100% |
| **Escherichia phage PNJ1809-36** | 292 | 152.343Kb | 39% | OL741059.1 | 94.3% |
| **Escherichia phage phAPEC8** | 274 | 147.737Kb | 39% | NC_020079 | 93.4% |
| **Dompiswa phage**  **TSP7_1** | 283 | 150.9 kb | 39% | NC_062742 | 92.8% |
| **Escherichia phage**  **Mt1B1_P17** | 295 | 151.02Kb | 39% | NC_052662 | 92.1% |
| **Escherichia phage**  **anhysbys** | 282 | 149.335Kb | 39% | NC_052656 | 90.8% |
| **Escherichia phage**  **vB_EcoM-Ro121lw** | 292 | 149.8Kb | 39% | MH160766 | 92.8% |
| **Klebsiella phage**  **ZCKP1** | 267 | 150.925Kb | 39% | NC_047994 | 89.4% |

**Table S10A; Genome similarity index computed by ViPTree software for SKA49 to generate phylogenetic tree**

| **S #** | **ID** | **length** | **taxid** | **name** | **group** | **score** | ***S*_G_** | **%.mean.idt** | **%.len** |
| --- | --- | --- | --- | --- | --- | --- | --- | --- | --- |
| **1** | **OL741059.1** | **51548** | **-** | **Escherichia phage SKA49** | **-** | **35354** | **1** | **100** | **99.7** |
| 2 | NC_048799 | 52329 | 2589658 | Escherichia phage Mangalitsa | dsDNA | 29649 | **0.8386** | 90.3 | 94.4 |
| 3 | NC_047830 | 53092 | 2005048 | Escherichia phage ST32 | dsDNA | 29583.5 | **0.8368** | 89.8 | 94.3 |
| 4 | NC_010106 | 52975 | 451705 | Escherichia phage phiEcoM-GJ1 | dsDNA | 28580 | **0.8084** | 87 | 94.3 |
| 5 | NC_048845 | 52092 | 2696397 | Escherichia phage flopper | dsDNA | 28511.5 | **0.8065** | 89.8 | 91.6 |
| 6 | NC_049466 | 52401 | 2601675 | Escherichia phage vB_EcoM_4HA13 | dsDNA | 18504.5 | **0.5234** | 70.9 | 72.8 |
| 7 | NC_047978 | 54065 | 2182346 | Erwinia phage Faunus | dsDNA | 16645.5 | **0.4708** | 66.9 | 70.3 |
| 8 | NC_023865 | 55098 | 1399915 | Pectobacterium phage PM1 | dsDNA | 15838 | **0.448** | 64.6 | 66.8 |
| 9 | NC_019504 | 56621 | 1051676 | Erwinia phage vB_EamM-Y2 | dsDNA | 15401 | **0.4356** | 63.4 | 65.8 |
| 10 | NC_047791 | 53333 | 1916414 | Pectobacterium phage PP101 | dsDNA | 15090.5 | **0.4268** | 64.7 | 66.1 |
| 11 | NC_048875 | 54982 | 2721760 | Pantoea phage vB_PagM_SSEM1 | dsDNA | 14936.5 | **0.4225** | 63.8 | 65.6 |
| 12 | NC_048803 | 53392 | 2650874 | Proteus phage Myduc | dsDNA | 13625 | **0.3854** | 59.3 | 63.6 |
| 13 | NC_025459 | 53744 | 1505227 | Aeromonas phage pAh6-C | dsDNA | 5317.5 | **0.1504** | 48.8 | 30 |
| 14 | NC_023594 | 54791 | 1445859 | Shewanella phage Spp001 | dsDNA | 5207 | **0.1473** | 46.2 | 31 |
| 15 | NC_047824 | 54319 | 1970795 | Shewanella phage SppYZU05 | dsDNA | 4952 | **0.1401** | 48.4 | 28 |
| 16 | KJ936628 | 50431 | 1524880 | Vibrio phage VPp1 | dsDNA | 1221.5 | **0.0346** | 50.4 | 6.2 |
| 17 | NC_031274 | 50509 | 1784982 | Pseudomonas phage O4 | dsDNA | 1058 | **0.0299** | 46.3 | 6 |
| 18 | NC_007808 | 49639 | 347327 | Pseudomonas phage PA11 | dsDNA | 1008 | **0.0285** | 47.2 | 5.7 |
| 19 | NC_020201 | 142349 | 1116482 | Pectobacterium phage phiTE | dsDNA | 956.5 | **0.0271** | 52.6 | 4.8 |
| 20 | NC_029065 | 98765 | 1729937 | Pseudomonas phage VCM | dsDNA | 927 | **0.0262** | 40.6 | 6.2 |
| 21 | NC_048861 | 90710 | 2712958 | Proteus phage Privateer | dsDNA | 909.5 | **0.0257** | 59.1 | 4.2 |
| 22 | NC_048664 | 92122 | 2699738 | Cronobacter phage vB_CsaP_009 | dsDNA | 887.5 | **0.0251** | 61.3 | 3.9 |
| 23 | NC_042054 | 92816 | 1777069 | Pseudomonas phage vB_PsyM_KIL4 | dsDNA | 882 | **0.0249** | 41.1 | 5.6 |
| 24 | NC_019540 | 49390 | 1161935 | Salinivibrio phage CW02 | dsDNA | 865.5 | **0.0245** | 45.9 | 5.2 |
| 25 | NC_031280 | 104906 | 1605379 | Acinetobacter phage vB_AbaM_phiAbaA1 | dsDNA | 856.5 | **0.0242** | 43.5 | 5.5 |
| 26 | NC_030934 | 90552 | 1777065 | Pseudomonas phage vB_PsyM_KIL1 | dsDNA | 835 | **0.0236** | 41.5 | 5.2 |
| 27 | NC_025462 | 99730 | 1481186 | Acinetobacter phage vB_AbaM_Acibel004 | dsDNA | 819 | **0.0232** | 43.4 | 5.3 |
| 28 | NC_023601 | 98287 | 1458843 | Pseudomonas phage phiPsa374 | dsDNA | 808.5 | **0.0229** | 40.4 | 5.4 |
| 29 | NC_025436 | 133824 | 1458859 | Shewanella sp. phage 1/4 | dsDNA | 754 | **0.0213** | 40.9 | 5 |
| 30 | NC_048074 | 103628 | 2315466 | Acinetobacter phage vB_AbaM_B09_Aci01-1 | dsDNA | 740.5 | **0.0209** | 42.2 | 5 |

**Table S10B; Genome similarity index for SKA64 and its related taxa used to generate phylogenetic tree**

| **Serial #** | **ID** | **length** | **taxid** | **name** | **group** | **score** | ***S*_G_** | **%.mean.idt** | **%.len** |
| --- | --- | --- | --- | --- | --- | --- | --- | --- | --- |
| **1** | **OM362897** | **152401** | **-** | **Escherichia Phage SKA64** | **-** | **102182** | **1** | **100** | **100** |
| 2 | NC_052654 | 151671 | 2144176 | Escherichia phage vB_EcoM-Ro121c4YLVW | dsDNA | 94069 | **0.9206** | 96.6 | 96.5 |
| 3 | NC_062742 | 150892 | 2793345 | Dompiswa phage TSP7_1 | dsDNA | 93782.5 | **0.9178** | 97.2 | 95.7 |
| 4 | NC_020079 | 147737 | 1229753 | Escherichia phage phAPEC8 | dsDNA | 93465 | **0.9147** | 97.6 | 94.4 |
| 5 | NC_047770 | 149813 | 1881104 | Escherichia phage ESCO13 | dsDNA | 93453 | **0.9146** | 96.7 | 95.5 |
| 6 | NC_048196 | 151194 | 2508195 | Escherichia phage vB_EcoM_Schickermooser | dsDNA | 93369.5 | **0.9138** | 96.2 | 95.9 |
| 7 | NC_052662 | 151202 | 2743961 | Escherichia phage Mt1B1_P17 | dsDNA | 93190 | **0.912** | 97.2 | 94.5 |
| 8 | NC_052660 | 150473 | 2696455 | Escherichia phage tuntematon | dsDNA | 92198.5 | **0.9023** | 97 | 93.6 |
| 9 | NC_052656 | 149335 | 2696383 | Escherichia phage anhysbys | dsDNA | 91439.5 | **0.8949** | 96.8 | 93.7 |
| 10 | NC_047994 | 150925 | 2201417 | Klebsiella phage ZCKP1 | dsDNA | 91207.5 | **0.8926** | 95.9 | 94 |
| 11 | NC_052661 | 150947 | 2696458 | Escherichia phage ukendt | dsDNA | 90926 | **0.8898** | 96.1 | 94 |
| 12 | NC_047776 | 149268 | 1897495 | Escherichia phage ESCO5 | dsDNA | 90722 | **0.8878** | 96.4 | 93.4 |
| 13 | NC_052658 | 151514 | 2696431 | Escherichia phage nepoznato | dsDNA | 88962.5 | **0.8706** | 96.1 | 91.6 |
| 14 | NC_052659 | 144998 | 2696432 | Escherichia phage nieznany | dsDNA | 86773.5 | **0.8492** | 96 | 89.1 |
| 15 | NC_052653 | 149506 | 2315527 | Escherichia phage vB_vPM_PD06 | dsDNA | 50171 | **0.491** | 69.8 | 68.9 |
| 16 | NC_052655 | 147009 | 2696379 | Escherichia phage alia | dsDNA | 49583 | **0.4852** | 69.1 | 68.6 |
| 17 | NC_052652 | 147659 | 2041347 | Escherichia phage vB_EcoM_PHB05 | dsDNA | 49412 | **0.4836** | 69.3 | 68.1 |
| 18 | NC_023693 | 148612 | 948870 | Escherichia phage phi92 | dsDNA | 49043 | **0.48** | 69 | 68.2 |
| 19 | NC_052657 | 146307 | 2696426 | Escherichia phage muut | dsDNA | 48392 | **0.4736** | 69.5 | 66.4 |
| 20 | NC_052663 | 147702 | 2776821 | Escherichia phage VEcB | dsDNA | 48343.5 | **0.4731** | 69.4 | 66.4 |
| 21 | NC_016071 | 145964 | 889338 | Salmonella phage PVPSE1 | dsDNA | 11654 | **0.1141** | 51.9 | 20.4 |
| 22 | NC_027351 | 147745 | 1204529 | Salmonella phage SSE121 | dsDNA | 11591 | **0.1134** | 51.9 | 20.3 |
| 23 | NC_022968 | 148567 | 1391428 | Escherichia phage 4MG | dsDNA | 10279.5 | **0.1006** | 50.2 | 18.6 |
| 24 | NC_019400 | 147940 | 1141135 | Cronobacter phage vB_CsaM_GAP31 | dsDNA | 9815 | **0.0961** | 50.4 | 17.9 |
| 25 | NC_028248 | 136896 | 1720495 | Escherichia phage slur16 | dsDNA | 9503.5 | **0.093** | 48.5 | 17.8 |
| 26 | NC_041871 | 135391 | 1675606 | Escherichia phage Murica | dsDNA | 9466.5 | **0.0926** | 48.2 | 17.9 |
| 27 | NC_024134 | 139020 | 1446490 | Escherichia phage FFH2 | dsDNA | 9448.5 | **0.0925** | 48.4 | 17.9 |
| 28 | NC_022323 | 136910 | 1327956 | Escherichia phage JES2013 | dsDNA | 9383.5 | **0.0918** | 49 | 17.5 |
| 29 | NC_041869 | 135400 | 1655314 | Escherichia phage APECc02 | dsDNA | 9315.5 | **0.0912** | 47.9 | 17.7 |
| 30 | NC_011041 | 137947 | 399183 | Escherichia phage V5 | dsDNA | 9284 | **0.0909** | 48.4 | 17.5 |
| 31 | NC_019517 | 136947 | 1131317 | Escherichia phage FV3 | dsDNA | 9259 | **0.0906** | 47.2 | 17.9 |
| 32 | KP869103 | 137973 | 1508680 | Escherichia coli O157 typing phage 5 | dsDNA | 9240 | **0.0904** | 48.5 | 17.4 |
| 33 | NC_041937 | 129454 | 1981500 | Escherichia phage V18 | dsDNA | 8879 | **0.0869** | 48 | 16.9 |
| 34 | NC_048656 | 147443 | 1907784 | Klebsiella phage vB_KpnM_BIS47 | dsDNA | 7548 | **0.0739** | 48 | 14.6 |
| 35 | NC_028659 | 142987 | 1719140 | Klebsiella phage vB_KpnM_KB57 | dsDNA | 7488 | **0.0733** | 47.7 | 14.6 |
| 36 | NC_048647 | 146203 | 1871716 | Klebsiella phage KNP2 | dsDNA | 7368.5 | **0.0721** | 47.6 | 14.4 |
| 37 | NC_048741 | 145127 | 2483610 | Proteus phage Mydo | dsDNA | 7211 | **0.0706** | 47.5 | 14.1 |
| 38 | NC_048873 | 143800 | 2847815 | Klebsiella phage KpS8 | dsDNA | 7204 | **0.0705** | 47.7 | 14 |
| 39 | NC_048682 | 145759 | 2053702 | Raoultella phage Ro1 | dsDNA | 7076.5 | **0.0693** | 47.3 | 13.9 |


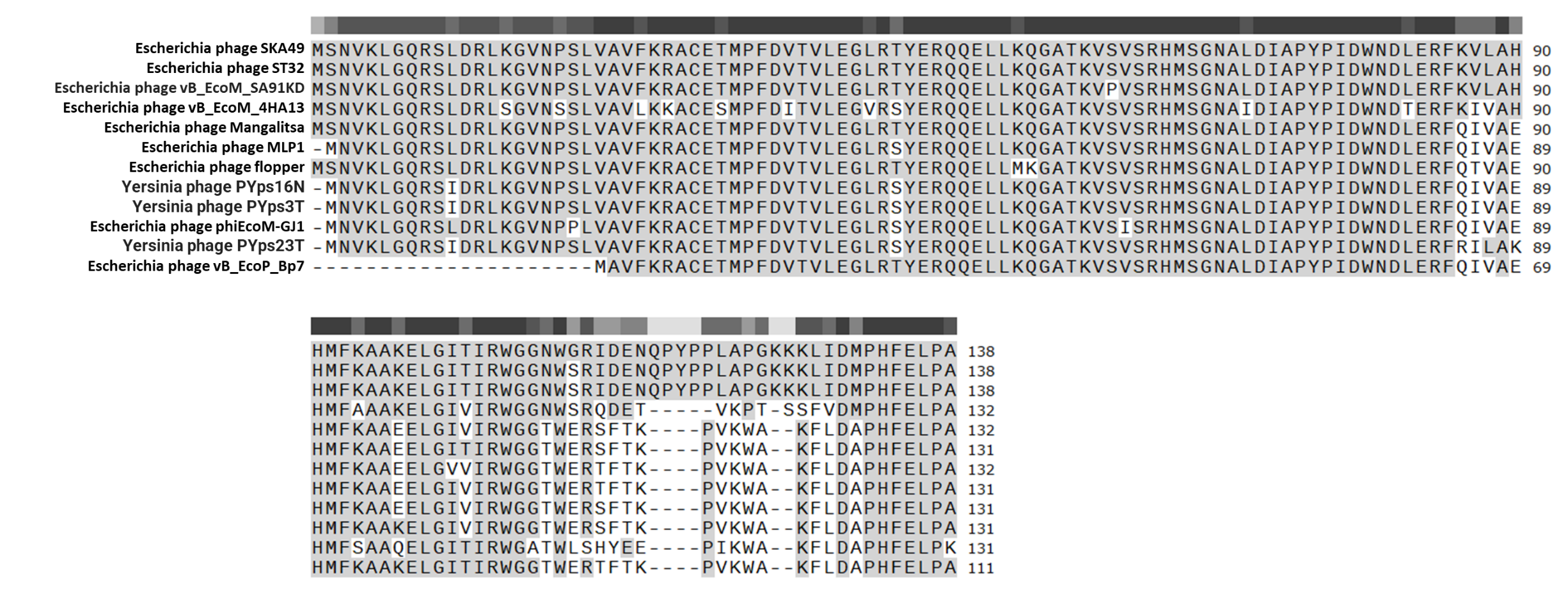


**Figure S3A. Amino acid sequence comparison of D-Alanyl-D glutamate Endolysin (ORF 22) from SKA49 with close homologs in BLASTp. N terminus of protein is conserved whereas C terminus has variation. Gray shading indicates residues that resemble SKA49 endolysin protein sequence.**


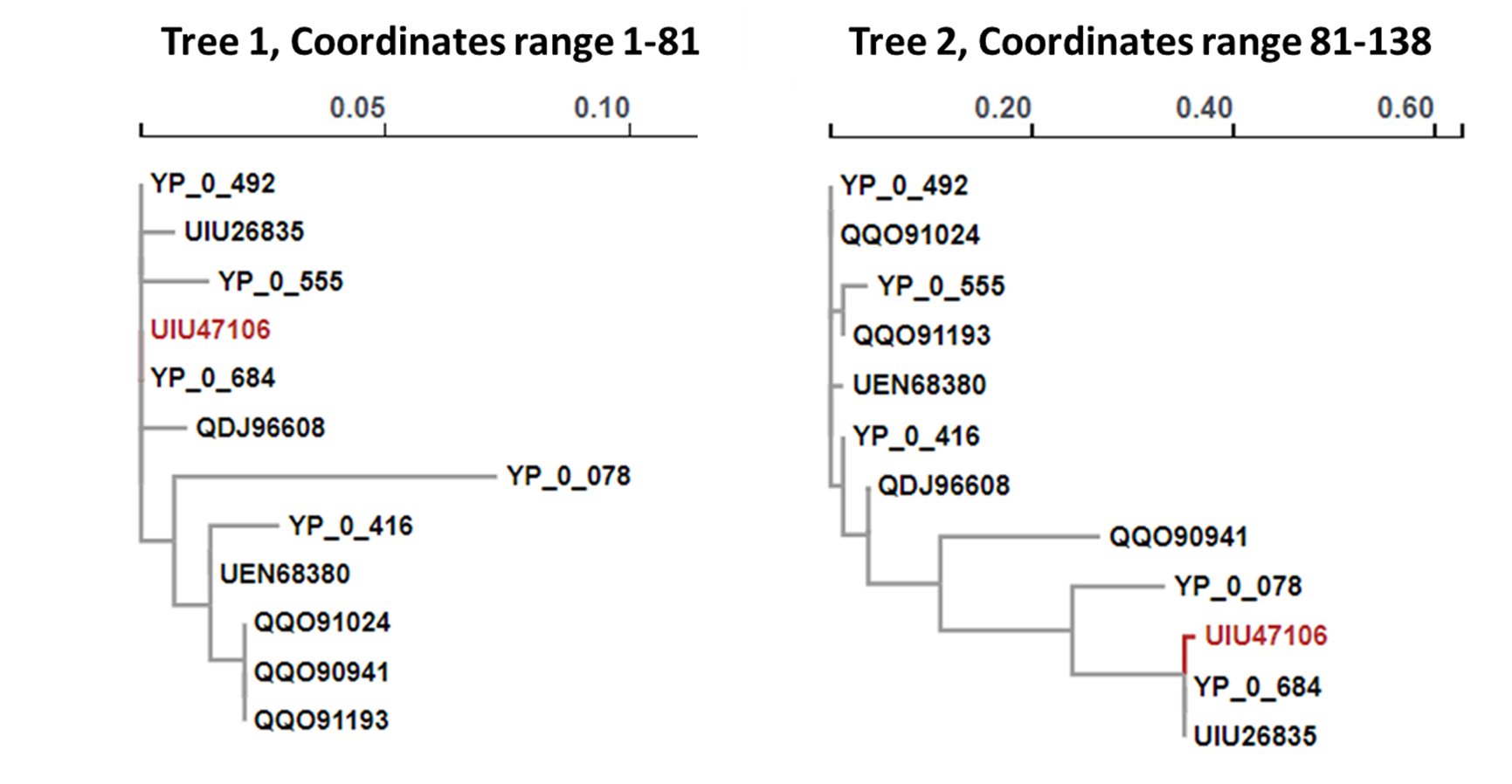


**Figure S3B. The GARD analysis of amino acid sequence of D-Alanyl-D-glutamate Endolysin (ORF 22) from SKA49. This analysis identifies a recombination hotspot at amino acid 81-82, which produces two distinct homology trees for N-terminus and C-terminus of protein indicating its chimeric nature.**

1. Bokhari H, Shah MA, Asad S, Akhtar S, Akram M, Wren BW. Escherichia coli pathotypes in Pakistan from consecutive floods in 2010 and 2011. American Journal of Tropical Medicine and Hygiene. 2013;88(3):519–25.

2. Hamid T, Wahab A dulaimi H, Ali A. Molecular Characterization and Antibiotic Susceptibility of Diarrheagenic Escherichia coli from Children Molecular Characterization and Antibiotic Susceptibility of Diarrheagenic Escherichia coli from Children College of Science for Women , Babylon Univer. 2015;(September 2016).

3. Hospitals C, Iqbal A, Mustafa MZ, Ahmad I, Rahman S. Incidence of Diarrheagenic. 2019;51(6):2015–21.

4. Kavitha K, Prabhakar K, Rajendran S, Uma B, Sarayu YL. Isolation of necrotoxigenic Escherichia coli from paediatric patients with acute diarrhoea. J Med Microbiol. 2010;59(4):503–4.

5. Nichols KB, Totsika M, Moriel DG, Lo AW, Yang J, Wurpel DJ, et al. Molecular characterization of the vacuolating autotransporter toxin in uropathogenic Escherichia coli. J Bacteriol. 2016;198(10):1487–98.

6. Jeong YW, Kim TE, Kim JH, Kwon HJ. Pathotyping avian pathogenic Escherichia coli strains in Korea. J Vet Sci. 2012;13(2):145–52.

7. López-Banda DA, Carrillo-Casas EM, Leyva-Leyva M, Orozco-Hoyuela G, Manjarrez-Hernández ÁH, Arroyo-Escalante S, et al. Identification of virulence factors genes in escherichia coli isolates from women with urinary tract infection in Mexico. Biomed Res Int. 2014;2014.

8. Clermont O, Christenson JK, Denamur E, Gordon DM. The Clermont Escherichia coli phylo-typing method revisited: Improvement of specificity and detection of new phylo-groups. Environ Microbiol Rep. 2013;5(1):58–65.
